# Facilitators and barriers of general health service readiness in the primary level public health facilities of Baitadi, Nepal: A qualitative study

**DOI:** 10.1371/journal.pgph.0006043

**Published:** 2026-02-23

**Authors:** Amrit Bist, Amit Arjyal, Madhusudan Subedi

**Affiliations:** School of Public Health, Patan Academy of Health Sciences, Lalitpur, Nepal; PLOS: Public Library of Science, UNITED STATES OF AMERICA

## Abstract

Health service readiness is crucial for the healthcare facilities to deliver quality care. Ensuring service readiness not only improves the quality of care but also enhances patient trust, service utilization, and overall health system performance, particularly in resource-constrained settings. The aim of this study was to explore the facilitators and barriers of general health service readiness in primary-level health facilities of Baitadi, Nepal. A cross-sectional study was conducted among the ten primary public health facilities of Baitadi, Nepal using a qualitative method. Ten key informant interviews were conducted with health facility in-charges. The inter-coder reliability was found 81.8% and Braun and Clarke’s thematic analysis was carried out for the analysis using RQDA software. Five themes and eleven subthemes were identified from the analysis. Facilities with low health service readiness commonly lacked adequate physical infrastructure, and faced frequent shortages of essential drugs and equipment. Limited trainings and irregular supervision further hindered readiness. Geographical barriers and procurement delays slowed the supply of resources, while regular staff meetings, supportive municipal leadership, and effective coordination emerged as key facilitators. Overall, the study highlights that improving coordination, leadership, and motivation of health service provider is essential for strengthening service readiness in resource-limited settings. Addressing persistent systemic obstacles such as difficult terrain, delays in supplies, and gaps in infrastructure remains equally important. Focusing on these priority areas through evidence-based and context-specific strategies can contribute to more responsive and higher-quality health services.

## Introduction

General Health Service Readiness reflects an ability of health facilities to efficiently deliver basic services, based on key domains such as basic amenities, equipment, infection prevention, diagnostics, and essential medicines [1,2]. It serves as a key indicator of a health system’s functionality in meeting population needs. Tools like the WHO and USAID’s Service Availability and Readiness Assessment (SARA) help monitor and evaluate this readiness, supporting evidence-based policy, equitable resource allocation, and system strengthening [2]. The readiness index also highlights disparities and measures how well health investments translate into service capacity. Ultimately, resilient health systems not only respond effectively in crises but also ensure consistent, quality care in normal times [1–3].

The performance of the health system is influenced by five main factors: organisation, payment, regulation, behaviour, and funding. Accessibility, effectiveness, and care quality are all impacted by the shifting dynamics of these five factors [4]. According to the WHO, the delivery of health services is one of the six pillars of the health system, and it necessitates accessibility, affordability, and acceptance of the services offered. Despite this, while preparedness and accessibility are essential for providing high-quality healthcare, they are insufficient on their own. In order to ensure fairness and improve population health, a robust health system should integrate them with strong mechanisms for delivering high-quality care [5].

The Sustainable Development Goals (SDGs) have emphasized universal health coverage (UHC) by promoting equitable access to health services. A key component of UHC is not just access, but the provision of high-quality care, which relies heavily on health service readiness. While global progress between 2000 and 2021 reduced the population at risk of poor access by 15%, momentum slowed after 2015, leaving 4.5 billion people still uncovered by 2021 [6]. Service readiness, part of the Donabedian quality framework, ensures the availability of essential inputs like infrastructure, equipment, medicines, and trained staff. However, true effectiveness depends on how these inputs are used to deliver patient-centered, timely, and efficient care. For UHC to be impactful, efforts must go beyond expanding services to also address care quality, provider competencies, and systemic barriers such as affordability, cultural acceptance, and geographic access. Without integrating quality, UHC efforts risk deepening health inequities and falling short of SDG goals [7,8].

Quality of care is a key component of health system strengthening, especially within the context of achieving Universal Health Coverage (UHC). Nepal underscores this through its Policy on Quality Assurance in Health Care Services (2064), which emphasizes setting and maintaining standards across all health sectors [9]. The Nepal Health Sector Strategy (2015–2020) further advocates for integrating quality at the service delivery level through a dedicated quality assurance body responsible for standard setting, enforcement, and monitoring [10]. Aligned with the National Health Policy 2019 and the Constitution of Nepal 2015, the government prioritizes equitable access to quality healthcare for all [11,12]. These efforts support Nepal’s commitment to UHC by ensuring that all citizens can access comprehensive, quality services without financial hardship. Addressing bottlenecks in health service readiness is crucial for Nepal to advance towards achieving universal health coverage (UHC) and reducing inequities in healthcare access and utilization [13].

However, despite these policy commitments, there is limited evidence on how health service readiness is shaped by local contextual factors particularly in geographically remote and underserved districts of Nepal. Existing studies largely focus on national or regional readiness scores mainly using quantitative approach. Moreover, little is known about how governance, infrastructure, supply chains, and human resources interact to influence readiness in these contexts which can be better known through qualitative insights. Addressing these gaps is essential for informing targeted strategies to strengthen service delivery and advance UHC in rural Nepal. This study explores the facilitators and barriers to general health service readiness in primary-level public health facilities in Baitadi, one of Nepal’s most remote hilly districts, using a qualitative approach. The findings will not only contribute to local health system strengthening but also offer insights that can be applied to similar contexts in other parts of Nepal and countries with comparable challenges.

## Materials and methods

### Study design and setting

A health facility based cross-sectional, inductive qualitative study design was carried out to explore facilitators and barriers of general health service readiness among the primary public health facilities in Baitadi district of Sudurpaschim Province, Nepal. The study employed qualitative method to provide a comprehensive understanding of the facilitators and barriers of health service readiness.

This study was conducted in Baitadi District, a remote hilly area in Sudurpaschim Province, Nepal, which has a Human Development Index (HDI) of 0.547, below the national average [14]. Baitadi, with 49,428 households and a literacy rate of 76.8%, faces significant health challenges despite ongoing efforts [15]. Institutional delivery in the district is 82%, lower than the provincial average of 93%, and it reported the second-highest neonatal and maternal mortality rates in the province in 2021/22 [16]. Baitadi is situated approximately 840 kilometers from Nepal’s capital, Kathmandu. Traveling this distance by public bus typically takes around 30 hours under normal conditions, and the journey can take considerably longer during the rainy season due to road blockages and landslides. The district has the difficult terrain, scattered population, and socio-economic constraints that might cause limited access to care and logistical challenges in delivering medical supplies [17].

### Sample size, sampling technique and data collection

The general health service readiness in Baitadi district was initially assessed across 68 primary-level health facilities to establish a baseline understanding before the qualitative component of the study. These facilities included five different types: Primary Health Care Centers (PHCCs), Health Posts (HPs), Basic Health Service Centers (BHSCs), Urban Health Centers (UHCs), and Community Health Units (CHUs). Based on the service readiness scores, key informants were purposively selected for further qualitative inquiry. Specifically, ten key informant interviews were conducted with the in-charges of health facilities representing the highest and lowest readiness scores within each facility type. The health facility in-charge are the head of facility responsible for overseeing and managing the health facility with service delivery and daily operations. The participants in the qualitative study were all males aged between 23 and 52 years and had professional experience ranging from 1 to 26 years. They represented a range of cadres within the primary health care system, including Public Health Inspectors, Health Assistants (HAs), Auxiliary Health Workers (AHWs), Senior AHWs, and Senior AHW Officers. Data saturation is reached after interviews with ten key informants and we stopped further collection of data. This approach aimed to capture a diverse range of perspectives regarding the factors influencing service readiness and operational challenges at the primary care level. Open ended standardized interview guideline was used to conduct KII for exploring facilitators and barriers of service readiness. Audio recordings and field note was taken during the interviews. The qualitative data was collected from 5 November, 2024 to 26 November, 2024 using the key interview guidelines with the in-charge of health facilities. The data supporting the findings are provided in the file (S1 Data).

### Trustworthiness of study

#### Credibility*.*

To ensure credibility, the interview guides were developed based on a comprehensive review of relevant literature. The draft tools were subsequently validated through consultations with subject experts, and revisions were made according to their feedback. During data collection, efforts were made to establish trust and rapport with participants to facilitate in-depth exploration of the facilitators and barriers influencing service readiness. The interviews employed iterative questioning to obtain rich, detailed information. In addition, frequent debriefing sessions were conducted among the research team to discuss emerging insights and interpretations. To further enhance credibility, the transcription and translation files were shared with team members for review, ensuring that the captured data accurately reflected participants’ responses.

#### Dependability.

To ensure the dependability of the generated codes in qualitative data analysis, inter-coder agreement was calculated. This process involved that two coders independently analyzed the same set of data and then compared their coding decisions to assess the level of agreement. The inter-coder reliability was found to be 81.8%, which is considered a strong level of agreement in qualitative research. A high percentage indicates that the coding process was systematic and replicable, reducing the likelihood of subjective bias in data interpretation. This reliability measure ensures that the final coding framework is robust, consistent, and accurately represents the data, ultimately strengthening the credibility and trustworthiness of the study’s findings.

#### Confirmability*.*

Researcher’s presumptions and beliefs were not used during the process of interview and data analysis. The researcher only acted as a facilitator during the interviews. When analyzing the data, the researcher has relied strictly on the participants’ responses and not allow personal biases or beliefs to influence the interpretation of the data. This involved maintaining transparency about the methods used for analysis and ensuring that interpretations were rooted in the data collected from participants. To enhance confirmability, researchers kept an audit trail that documents decisions made during the research process. This included detailed records of how data were collected, how themes were developed, and how conclusions were reached. This allowed others to follow the reasoning behind the findings and assess the objectivity of the study.

#### Transferability.

Background information was included to establish the study’s context and to make readers familiar with the context of the study. This included details about the setting, participants, and the conditions under which the study was conducted. The provided rich, detailed descriptions of the research context, participants, and findings will allow readers to determine the applicability of the results to their own settings.

### Positionality and reflexivity

The study site being the home district of the main author, facilitated coordination and support during data collection. Similarly, the PI’s involvement in the Nepal Public Health Association, a professional body for public health graduates, helped for the coordination and rapport building. Throughout the research, researcher was aware about how backgrounds and experiences might affect research work. Researcher was aware of personal views and stayed aware during the data collection process.

### Data analysis

The qualitative interviews were audio-recorded using the researcher’s mobile phone and later transferred to a password-protected computer for security. The recordings were transcribed into Nepali and then translated into English by the researcher, with verification by the research guide. Further, the translated files were converted to text format for import into the RQDA (R Package for Qualitative Data Analysis) software for coding and categorization of code. Qualitative data from key informant interviews were analyzed under thematic analysis following Braun and Clarke’s six-step framework. Presentation of findings is done based on themes and subthemes with appropriate verbatim. The themes and sub-themes are provided in Table 1.

**Table 1:** Theme and sub-themes of study.

Themes	Sub-themes
Strengthened Governance and Leadership Practices	Effective coordination
	Supportive leadership
Workforce Capacity and Performance Support	Staff trainings
	Regular supervision
Health Facility Infrastructure and Supplies	Physical infrastructure
	Resource availability
Environmental and Operational Constraints	Geographical difficulties
	Delay in procurement
Management Processes and Staff Engagement	Routine staff meeting
	Routine HFOMC Meeting
	staff motivation

### Ethical approval

All methods of this study were carried out under the Helsinki Declaration of ethical principles for medical research involving human subjects. Ethical approval was obtained from Institutional Review Committee (IRC) of Patan Academy of Health Sciences (Ref: PHP2409201926) before starting the field work. The formal approval of study was obtained from Health Office, Baitadi (Ref: 2081/2082-16). Information sheet was provided to the participants and written informed consent was taken from all the participants after detail explaining the study. Along with this, the participants were assured of their anonymity and confidentiality of the findings, with voluntary participation and right to withdraw at any point of time without giving any reason during study period or interview. The data storage was done in researcher’s laptop which remained confidential with password security.

### Inclusivity in global research

Additional information regarding the ethical, cultural, and scientific considerations specific to inclusivity in global research is included in the Supporting Information. (S1 Checklist)

## Results

### Demographic information of the participants

The study participants in the qualitative study were aged between 23 and 52 years, with varying levels of professional experience ranging from 1 to 26 years. Their roles in the health sector included Public Health Inspectors, Health Assistants (HA), Auxiliary Health Workers (AHWs), Senior AHWs, and Senior AHW Officers. The majority had several years of experience in their respective fields, with more senior positions generally associated with longer work experience. This diverse mix of health professionals provides valuable insights into their perspectives based on their varying levels of expertise and service duration.

### Themes and sub-themes

The data were analyzed using the Braun and clerk six steps thematic analysis where following themes and sub themes were identified (Table 1).

### Theme: Strengthened governance and leadership practices

#### Sub-theme: Effective coordination.

Based on the interview, it was explored that timely coordination with the municipality was critical in addressing resource and logistical challenges. It facilitated smoother supply of essential medicines, equipment, and other resources without delays. By ensuring that resources were readily available and distributed efficiently, the coordinated efforts between staff and the municipality significantly enhanced the overall readiness and responsiveness of health services, ultimately improving patient care. A key informant mentioned, *“One cannot achieve this alone. Good coordination is essential. It is not enough for health workers to work hard alone. The availability of essential medicines, equipment, and other resources depends on coordination. Our coordination with the municipality and ward is excellent. They have been very supportive in all aspects.”,* 23 years, KII_3

Another interviewee mentioned that, “*Our coordination is quite good. The required resources have been made available to us, which has positively contributed to readiness.*”, 38 years, KII_4

#### Sub-theme: Supportive leadership.

Our findings indicated that active municipal leadership was another essential facilitator for the service readiness. Supportive leaders not only prioritized improvement in the health sector but also secured the necessary resources and facilitated prompt decision-making. This team approach among staff, coordination and leadership greatly contributed to the readiness of health services. A key informant mentioned, “*In some places, leadership pays little attention to health, focusing instead on other plans. But here, the municipal and ward chairs are actively involved. It is not just about supplying medicines, buildings are also important, including for PHC/ORC services. Leadership support is crucial for such infrastructure. Here, the leadership has been very supportive whenever needed.*”, 23 years, KII_3

Another key informant also explained as, “*We staff hold discussions about how to improve the health unit. For problem-solving, we communicate with the health section and the chairperson. They respond positively and are proactive in addressing the issues we bring to their attention.*”, 33 years, KII_5

### Theme: Workforce capacity and performance support

#### Sub-theme: Staff training.

Training played a crucial role in improving service readiness in the health facilities securing high readiness score. The findings revealed that municipality identifies training needs and ensures staff receive necessary training. The training enhances skills, addresses shortcomings, and facilitates behavior change, especially for new staff with limited experience which ultimately helping for enhancing the readiness. A participant highlighted, “*The municipality identifies which staff members require what type of training and what training they have already received. Based on this, arrangements are made. Compared to other health institutions, staff at primary health centers receive more training opportunities, which has been helpful.”,* 42 years, KII_1

Another key informant said, “*We ensure that untrained staff attend the training. Periodic refresher training is also necessary. Trained staff can address shortcomings and deliver better services, ultimately improving readiness*.”, 37 years, KII_2

On the other hand, it was explored that inadequate training has created a challenge in enhancing the health service readiness. Limited participation in training programs, with the same staff attending repeatedly, hinders overall capacity building. An interviewee mentioned, “*Trainings have been conducted, but it would be better if all staff members participated in the process. For example, there are malaria and mental health training programs. It would be good if everyone could attend compulsory training. If the same people are trained each year, it can be difficult to provide effective services. Each year, different people should be trained.”*, 50 years, KII_10

Based on the interview, it was found that health workers from some facilities receive few or no training opportunities, making it difficult to improve readiness. An interview reflects on it as, “*In our Rural Municipality, the positions seem to be fulfilled so far. However, when there is a training program, it is limited to two participants from the municipality. We have not received even one training session this year. If staff members who have not had the chance to participate in training could be included, it would make improving readiness much easier. It is not sufficient for just one person to receive training; everyone should be provided training as per their needs.”,* 40 years, KII_6

Additionally, study revealed that the quality of training has declined, as expert trainers from outside are no longer involved, reducing its effectiveness. An interviewee stated, “*Training helps with areas we’re unfamiliar with. Trainings are conducted, but there’s a difference between the old and new trainings. Previously, expert trainers from outside used to come, but now trainers from within the district conduct them. Sometimes I provide, and other times someone else does. The quality of the training isn’t as it used to be, and it feels inadequate.*”, 52 years, KII_9

#### Sub-theme: Regular supervision.

Key informant interviews revealed that regular supervision enhances health service readiness through regular guidance, follow-up, and feedback to the health workers. A study participant mentioned, *“Supervision also occurs regularly, both from the municipality and the health office. When they visit, they come with a proper plan, which we follow to improve readiness. That might be why our readiness has improved.”*, 37 years, KII_2

Based on interview, it is evident that supervisors are helpful in facilitating resources, adherence to protocols, and increasing the motivation and accountability of the staff. An interviewee stated, *“Supervision has been very helpful. Just recently, officials from the health office visited us. A public health inspector from the district and the municipality chairperson also visited. They observed how we deliver services and identified any gaps. When they personally visit and see the shortcomings, they address them and provide supplies. This also motivates the staff.”*, 23 years, KII_3

On the other hand, findings showed that facilities with limited supervision from higher authorities has found lower readiness of health services. Most key informants from these facilities emphasized that increased supervisory visits could improve their readiness through better support and guidance, ensuring proper resource use. A key informant mentioned, “*This health institution is closest to the local government office, yet we have not received sufficient supervision. If higher authorities were to come for regular supervisory visits and provide supportive supervision on logistics, human resources, and staff matters, it would boost the morale of the staff. This would increase readiness and, in turn, directly benefit the service users.”*, 35 years, KII_7

### Theme: Health facility infrastructure and supplies

#### Sub-theme: Physical infrastructure.

This study showed that health facilities with high readiness were found to have better physical infrastructure. The adequate physical infrastructure with adequate rooms helped to maintain the audio-visual privacy of the patients and provide quality health services. An interviewee mentioned, “*The availability of physical infrastructure has significantly contributed to improving readiness. It plays a crucial role in managing other aspects of the facility.*”,37 years, KII_2

The findings revealed that health facilities with low readiness struggle with poor physical infrastructure. A lack of adequate buildings and space is a major barrier, affecting service readiness. A key informant said, “*We need a building to increase readiness. The lack of space is the biggest issue.*”, 50 years, KII_10

Some health facility rooms in Baitadi lacked adequate space and ventilation, and also faced issues of water leakage from the roof during the rainy season. Based on interview, it was found that facilities require proper rooms with sufficient spaces, ventilation and roofing. An interviewee said, “*We urgently need a proper building with sufficient rooms and ventilation, a delivery room with proper roofing, and equipment like refrigerators*.”, 40 years, KII_6

The absence of dedicated spaces for key services, such as autoclaving, OPD, and maternal care, limits compliance with standards. Establishing well-equipped buildings with designated service areas could significantly improve readiness and overall service quality. A participant said, “*The lack of infrastructure, such as buildings, is a major barrier. For example, we need a separate room for autoclaving as per MSS, but we don’t have space. Even temporary solutions like tin sheds aren’t feasible. Despite motivated and experienced staff, the lack of infrastructure hampers progress. Without our own building, it is difficult to manage many things. A building would solve many issues. With a proper building, we can have separate spaces for OPD, safe motherhood services, family planning, and medicine distribution, which would improve our scores.*”, 50 years, KII_8

However, the support from NGO/INGOs was found in enhancing the readiness related to the physical infrastructure in some of the health facilities having higher readiness score. An in-charge of health facility having high readiness score mentioned, “*NGOs/INGOs have supported us. An NGO helped improve the delivery room. They installed tiles, built an attached toilet and bathroom, set up water taps, and installed a water tank*.”, 37 years, KII_2

#### Sub-theme: Resource availability.

The study showed that facilities with high readiness have a better supply of equipment and resources. Based on interviews, it was evident that municipalities played a crucial role in promptly providing necessary medicines and equipment based on facility requests. An interview mentioned, “*The municipality has been supplying us with the required medicines and equipment as per our requests. First of all, I want to thank the municipality, as it has played a key role. We also have our role, whenever we identify any shortcomings, we immediately make a request, and they supply the needed resources promptly*.”,23 years, KII_3

However, some of the health facilities often lacks patient beds. An interviewee said, “*We didn’t even have beds for patients here. Just a few days ago, the municipality provided them. There is a lack of rooms, so we are managing by creating partitions. Recently, the ward allocated some funds, but we don’t even have curtains. We received a low score because we lack basic items like pillows under the minimum service standards.*”, 52 years, KII_8

Similarly, this study revealed that facilities with high readiness had reliable internet access which was facilitating online meetings and digital communication. Internet access also plays a crucial role in timely and accurate data recording and reporting. A healthy facility in-charge mentioned, “*It has definitely been beneficial. We have NT Fiber internet here, which is both affordable and fast. Previously, we used Everest’s internet, which was neither fast nor cost-effective. We used to pay NPR 3,300 per month for Everest internet, but now with NT Fiber, we manage with NPR 1,000 per month. The good network makes Zoom meetings and other online activities easier.*”, 42 years, KII_1

Another participant said, “*Internet and Wi-Fi have been immensely helpful. They enable timely and accurate recording and reporting. Without the internet, proper data management would not be possible. The internet has supported the eLMIS system, and implementing eHMIS would further improve efficiency*.”, 37 years, KII_2

However, there was a lack of equipment like laptops and dedicated internet connections in health facilities of Baitadi which further hampers communication and ultimately readiness score. These limitations affect data management, reporting, and overall service efficiency, and thereby reducing the service readiness. A key informant said, “*We don’t have a laptop, but we have requested one at the municipal level. We also do not have our own internet, but they are positive and say they will provide it.*”, 50 years, KII_10

Another study participant said, “*One problem is the network connectivity. At times, we cannot even make a call when needed. The network works a bit when there is sunny day, but once the sun sets, it goes off. Even on cloudy days, there is no network*.”, 33 years, KII_9

Key informant interviews identified some of the issues confronting primary-level health facilities’ diagnostic capacity, including insufficient resources that restrict comprehensive diagnostic services, where patients are forced to seek external initial diagnosis and return only for follow-ups and drugs. Supply chain issues, such as stock out of glucometer test strips and seasonal supply of malaria RDT kits, also weaken services. Absence of laboratory facilities in some of the health posts and irregular 32 supply of basic kits, like pregnancy tests for urine. These systemic issues severely undermine the readiness of these facilities to deliver timely and effective diagnostic care. An interviewee stated, “*Due to limited resources, we cannot perform all types of diagnoses. While initial diagnoses are conducted elsewhere, patients often visit us for follow-ups and to obtain necessary medicines*.”, 42 years, KII_1

Another participant said, “*Diagnostic tools like X-ray are not available at health post level and we receive rapid diagnostic kits for Malaria during its peak spread time only*.”, 52 years, KII_8

Findings revealed that shortage of essential medicines is a key barrier to health service readiness. Inadequate distribution of medicines results in insufficient stock at some facilities, limiting their ability to meet patient demand. Health facilities serving thousands of patients annually, often experience stock outs, leading to service disruptions and patient complaints. A key informant said, “*Medicines are not directly supplied to this facility from the municipality; they go through Gokuleshwor Health Post, which then distributes them to multiple facilities from there. For instance, if 500 tablets arrive, they need to be divided among three facilities, leaving us with insufficient stock to meet the needs of the large number of clients here.*”, 40 years, KII_6

Another participant mentioned, “*Our health institution receives approximately 13,000 to 15,000 patients annually. This is the second busiest health facility in the district, after the district hospital in Baitadi. We now face a significant need for logistics compared to other health 34 institutions. There are number of times when essential medicines and other logistics required for patient care run out of stock. This leads to situations where we have to listen to complaints from service users as well.*”, 35 years, KII_7

One of the health facility in-charge said that demand-based supply approach could improve availability and reduce wastage, ensuring facilities receive the medicines they need. “*Not all medicines are needed everywhere; for example, certain medicines might not be required in Surnaya rural municipality but are necessary in primary health centers with advanced services like X-ray facilities. Medicine supply should be based on the specific needs of each health institution, ensuring optimal utilization and minimizing wastage. Push systems often result in medicines expiring unused. Last year, atenolol 50 mg had to be discarded due to lack of demand. The health office should focus on demand-based supply*.”, 42 years, KII_1

However, the support from NGO/INGOs was found in enhancing the readiness in some of the health facilities having higher readiness score. One of the participants said, “*Once, an organization provided us with an inverter*.”, 38 years, KII_4

Another interviewee highlighted that, “*Recently an organization called NEEDS provided plumbing for our water supply, and UNFPA contributed freezers for vaccine storage. Many organizations have directly or indirectly supported us to make the facility more effective and efficient.*”, 35 years, KII_7

The interview revealed that lack of resources sometimes hinders the ability to provide the desired level of care, leading to feelings of inadequacy. Despite these challenges, staff remain committed to their duty, viewing service as a lifelong responsibility to help those in need. An interviewee said, “*I have worked in many places in the course of my career. I have also worked at the district level and had the opportunity to learn from doctors. I am very happy internally to be able to work at the community level and in the operation theater as well. However, it saddens me when I am unable to provide the services I want due to a lack of resources. It makes me feel inadequate. But as long as I live, I believe that service is a duty, and I will continue to work with responsibility to heal the wounds of the suffering.*” 50 years, KII_10

### Theme: Environmental and operational constraints

#### Sub-theme: Geographical difficulties.

Baitadi is located at 840 Kilometers from the capital city of Nepal, Kathmandu taking nearly 30 hours to reach by a public bus. Local road connectivity within the district is also poor, and many health facilities remain inaccessible by road. In our interviews, geographical remoteness emerged as a key factor hindering supply chains and health service readiness. One interviewee explained, “*the geographical challenges affect readiness due to limited physical infrastructure and human resources. The transportation of goods is also difficult due to the distance. We send request forms and have to carry the medicines ourselves. If it’s difficult to transport the supplies, we even use tractors to bring them. These factors impact readiness.*”, 50 years, KII_10

Another participant said, “*The main issue is geographical remoteness. If there was consistent transport and communication infrastructure, it would be much easier to manage. The transport needs to be available year-round, and the communication network should work 24/7. That would make everything much smoother.”,* 33 years, KII_9

One of participant highlighted that the health facility even doesn’t has its own land. He said, “*The health post doesn’t have road access. Transporting supplies is challenging. Additionally, the land doesn’t belong to us. However, a donor has proposed donating land near the road, and we’re moving forward with that process. Hopefully, it will be completed in a couple of months*.”, 52 years, KII_8

However, those health facilities which are located in better road access in Baitadi has better readiness. An interviewee of health facility located in urban area of district mentioned, “*Definitely. Geographical and transportation accessibility have been beneficial. If we face a shortage of supplies, they can be delivered from even Kathmandu or Dhangadhi within a day or two, which is certainly advantageous.”,* 42 years, KII_1

It was found that good geographical and transportation accessibility significantly enhances health service readiness by ensuring timely supply and regular supervisory visits. Facilities near roads experience smoother medicine distribution, with emergency supplies procured within hours and shortages addressed quickly, even from distant locations. An interviewee stated, “*Since the facility is located near the road, it has been easier to bring in delivery cases and improve other indicators. Municipality officials visit frequently, and we can easily communicate our issues. Coordination has also been facilitated. Medicine supply is smooth as we can safely transport medicines at any time.*”, 37 years, KII_2

#### Sub-theme: Procurement delays.

Based on findings, it is evident that delays in medicine procurement significantly impact health service readiness. The municipality frequently postpones tender processes leading to prolonged shortages. A participant said, “*Delays in medicine procurement are major reasons. Whether this is due to political reasons or staff inefficiency, we are not sure. The municipality is well aware that the Community Health Unit in Pujarigaun has the highest number of service users and institutional deliveries. Despite knowing this, the supply of medicines here is insufficient.”*, 40 years, KII_6

Key informants reported that medicines often arrive months late, with some facilities not receiving supplies since July. An interviewee reported, “*We haven’t received any medicines since the month of July. That’s why there is a shortage of medicines. When we talk to the municipality about it, they say that medicines come late because of delays in the procurement process. The medicines that were supposed to arrive by the end of June have not come yet.”,* 33 years, KII_9

Fragmented supply deliveries in multiple lots further compromise quality and timeliness. A key informant said, *“Delays happen because the municipality doesn’t issue tenders on time. They wait for a convenient time or budget adjustments, which delays the process. This year, they could have tendered in Shrawan (July-August) but did it only in Kartik (October-November). Additionally, the company provided medicines into three lots, which compromises the quality of medicines and further delays their supply.*”, 52 years, KII_8

### Theme: Management processes and staff engagement

#### Sub-theme: Routine staff meeting.

Based on interview, it was found that routine staff meetings provided opportunities for open communication, collaboration, and problem-solving among the healthcare workers. The meetings helped them discuss challenges, share updates, and align on ways to enhance the readiness and delivery of services. A study participant said, “*In staff meetings, we discuss the standards required for service delivery. This includes recording, reporting, and reviewing indicators. We meet monthly and, if necessary, twice a month to discuss our progress and plan future actions. This ultimately helps in readiness.*”, 37 years, KII_2

#### Sub-theme: Routine HFOMC meeting.

This study showed that routine HFOMC meetings play a crucial role in improving health service readiness by facilitating communication, prioritizing issues, and coordinating with relevant authorities. These meetings provide a platform for staff to raise concerns, discuss facility challenges, and develop action plans. The committee’s guidance and support help address logistical and operational gaps, ensuring that necessary resources and interventions are implemented effectively that strengthens the overall readiness of health facilities. A key informant stated, “*Issues discussed in staff meetings are forwarded to the management committee, which understands the priorities and plays a key role in improving readiness.”*, 37 years, KII_2

Another participant said, “*Management committee meetings are also held periodically where we present health facility problems and create plans.”,* 23 years, KII_3

#### Sub-theme: staff motivation.

Based on the interview, it was explored that staff motivation helped for health service readiness by fostering teamwork, efficiency, and a proactive work culture. Incentives, such as financial rewards and public recognition, boost morale and encourage better performance. An interviewee state, “*We have received incentives, such as a reward of NPR 5,000 from the municipality for good work. The ward also recognizes one volunteer and one staff member annually for their excellent work. These initiatives have boosted staff morale and motivated us to perform better, contributing to improved readiness.*”, 37 years, KII_2

This study showed that the supportive and collaborative work environment, where all staff regardless of contract status feel valued and work together, further strengthens service delivery. A key informant mentioned, “*Motivation brings new energy. It does not just improve readiness; it enhances everything. Even for contract staff, we encourage teamwork, telling them that being on contract does not matter. Everyone supports each other, and the staff here are motivated, which also contributes to readiness.*”, 23 years, KII_3

## Discussion

This study highlights several key factors influencing health service readiness at the primary care level in Baitadi district. This study identified coordination as a key facilitator of health service readiness, highlighting the importance of effective collaboration among stakeholders including health facility in-charges, local authorities, community people and partner organizations to streamline service delivery and resource utilization. Coordination can also foster a shared understanding of priorities, reduce duplication of efforts, and enhance accountability within the health system. In the context of Baitadi, where geographic inaccessibility and limited infrastructure pose significant challenges, strong coordination may be particularly crucial to ensure timely delivery of supplies, supervision, and support to remote health facilities. The finding aligns with a systematic review on integrating mental health into primary care, which also emphasized coordination as a critical enabler [18]. Supportive leadership emerged as another significant facilitator in this study. Strong leadership was shown to play a crucial role in motivating health workers, ensuring the timely allocation of resources, and fostering a positive and efficient work environment. This suggests that leadership is not merely an administrative function but a strategic factor that can shape the overall functionality of primary health care services. These findings mirror those of studies from South Carolina and Canada, where leadership support was directly linked to improved service readiness and better health outcomes [19,20]. In contrast, evidence from Nigeria and Uganda illustrates how poor leadership and lack of political will can act as major obstacles to service readiness. Specifically, inadequate governance hindered the delivery of Adolescent and Youth-Friendly Health Services in Nigeria and geriatric care in Uganda, emphasizing the importance of political commitment and good governance in health system strengthening [21,22]. In Baitadi, where local health governance is challenged by logistical constraints, supportive leadership appears to be a key determinant in mitigating structural barriers and maintaining service continuity.

Staff training and supervision were identified as critical enablers of service readiness. Regular capacity-building activities not only enhance the technical competence of health workers but also reinforce adherence to protocols and promote confidence in service delivery. Inadequate training or inconsistent supervision can lead to gaps in service quality, reduced efficiency, and missed opportunities for preventive care. These findings are consistent with prior research in low- and middle-income countries, which highlights training, supervision, and supportive mentorship as essential for maintaining primary health care readiness [23,24]. Likewise, poor infrastructure was echoed as a barrier in a study on age-friendly health services in Nigeria [25]. In the context of Baitadi, the combined impact of poor infrastructure, logistical challenges, and seasonal inaccessibility exacerbates these barriers, highlighting the need for strategic investments in both physical infrastructure and logistical resources. Taken together, these findings suggest that improving health service readiness requires an integrated approach that addresses both systemic and operational factors. Facilitators such as coordination, supportive leadership, and continuous training can partially mitigate structural challenges, while targeted investments in infrastructure and supply chain management are critical for sustainable improvements. Importantly, these insights underscore that health system strengthening is context-dependent; interventions must consider local geographic, political, and resource realities to be effective. This study was confined to primary level public health facilities, which may not accurately its facilitators and barriers in higher level facilities and private health facilities. These limitations highlight areas for further research to provide a more comprehensive understanding of health service readiness, its facilitators and barriers across diverse settings. This study explored facilitators and barriers in primary-level public health facilities. However, future research should be expanded to higher-level hospitals and private healthcare facilities to compare readiness, facilitators, and barriers across different healthcare settings. The insights of higher level health officials and political leadership can add more value. This study focused on health facility perspectives, but there is a need to examine the perceptions of community members and policymakers regarding health facility readiness, existing facilitators, and barriers. Understanding community expectations and experiences can help identify gaps in service delivery, while insights from policymakers can shed light on resource allocation, governance, and policy challenges. Such extended research will contribute to an even more holistic understanding of health service readiness, with evidence-based intervention and policy enhancement toward better healthcare service delivery.

## Conclusions

The qualitative finding revealed that effective coordination, supportive leadership, regular supervision, access to infrastructure and resources as facilitators while geographical difficulties and procurement delay, poor coordination, inadequate supervision as the major barriers for general health service readiness. Enhancing general health service readiness requires a multifaceted approach, including infrastructure development, improved coordination, regular supervision, and need-based training programs. Timely procurement and budget allocation can address supply chain issues, while aligning medicine supply with demand and promoting public-private collaboration can improve resource utilization.

## Supporting information

S1 Checklist Inclusivity questionnaire. (DOCX)

S1 Data Data. (DOCX)

## References

[1] Ghimire U, Shrestha N, Adhikari B, Mehata S, Pokharel Y, Mishra SR. Health system’s readiness to provide cardiovascular, diabetes and chronic respiratory disease related services in Nepal: analysis using 2015 health facility survey. BMC Public Health. 2020;20(1):1163. doi: 10.1186/s12889-020-09279-z 32711487 PMC7382840

[2] World Health Organization. Service availability and readiness assessment (SARA): an annual monitoring system for service delivery: reference manual. World Health Organization. 2013. https://iris.who.int/bitstream/handle/10665/149025/WHO_HIS_HSI_2014.5_eng.pdf?sequence=1

[3] Kruk ME, Myers M, Varpilah ST, Dahn BT. What is a resilient health system? Lessons from Ebola. Lancet. 2015;385(9980):1910–2. doi: 10.1016/S0140-6736(15)60755-3 25987159

[4] Roberts M, Hsiao W, Berman P, Reich M. Getting health reform right: a guide to improving performance and equity. Oxford: Oxford University Press. 2003.

[5] World Health Organization. Monitoring the building blocks of health systems: a handbook of indicators and their measurement strategies. Geneva: World Health Organization. 2010.

[6] United Nations. Ensure healthy lives and promote well-being for all at all ages. https://sdgs.un.org/goals/goal3#progress_and_info

[7] Donabedian A. The quality of care. How can it be assessed?. JAMA. 1988;260(12):1743–8. doi: 10.1001/jama.260.12.1743 3045356

[8] Acharya K, Paudel YR. General health service readiness and its association with the facility level indicators among primary health care centers and hospitals in Nepal. J Glob Heal Reports. 2019;3. doi: 10.29392/joghr.3.e2019057

[9] Ministry of Health and Population (MoHP) G of N. Policy on Quality Assurance in Health Care Services. Kathmandu: Ministry of Health and Population. 2007. https://mohp.gov.np/uploads/Resources/1657877771531Policy%20on%20Quality%20Assurance%20in%20Health%20Care%20Services.pdf

[10] Ministry of Health and Population (MoHP) G of N. Nepal Health Sector Strategy 2015–2020. Kathmandu: Ministry of Health and Population. 2015. https://www.nhssp.org.np/NHSSP_Archives/health_policy/NHSS_english_book_2015.pdf

[11] Ministry of Health and Population (MoHP) G of N. National Health Policy. Kathmandu: Ministry of Health and Population. 2019. https://mohp.gov.np/resources/plans-policies-acts-and-rules/en

[12] Constitution of Nepal 2015. 2015. http://www.lawcommission.gov.np/en/archives/category/documents/prevailing-law/constitution/constitution-of-nepal

[13] World Health Organization, South-East Asia Regional Office. Universal Health Coverage. 2024. https://www.who.int/southeastasia/health-topics/universal-health-coverage

[14] Central Bureau of Statistics. Census Nepal results. https://censusnepal.cbs.gov.np/results

[15] Nepal Human Development Report. UNDP. 2020. www.npc.gov.np

[16] Health Directorate SP. Provincial annual health report. Doti: Sudurpashchim Province Government. https://hd.sudurpashchim.gov.np/content/11122/11122-provincial-annual-health-repor/

[17] Maharjan KL, Joshi NP. A poverty analysis in Baitadi district, rural far western hills of Nepal: An inequality decomposition analysis. 2008. https://mpra.ub.uni-muenchen.de/35384/1/A_Poverty_Analysis_in_Baitadi_District_Rural_Far_Western_Hills_of_Nepal_An_Inequality_Decomposition_Analysis.pdf

[18] Addington D, Kyle T, Desai S, Wang J. Facilitators and barriers to implementing quality measurement in primary mental health care: Systematic review. Can Fam Physician. 2010;56(12):1322–31. 21375065 PMC3001932

[19] Zapka J, Simpson K, Hiott L, Langston L, Fakhry S, Ford D. A mixed methods descriptive investigation of readiness to change in rural hospitals participating in a tele-critical care intervention. BMC Health Serv Res. 2013;13:33. doi: 10.1186/1472-6963-13-33 23360332 PMC3565938

[20] Manalili K, Scott CM, O’Beirne M, Hemmelgarn BR, Santana M-J. Informing the implementation and use of person-centred quality indicators: a mixed methods study on the readiness, barriers and facilitators to implementation in Canada. BMJ Open. 2022;12(8):e060441. doi: 10.1136/bmjopen-2021-060441 36008077 PMC9422875

[21] Ladi-Akinyemi T, Balogun M, Welch S, Emmanuel G, Montalto F, Montalto G. Facility Preparedness, Barriers, and Facilitators to the integration and implementation of Adolescent and Youth-friendly health services in Primary Health Care centres in Southwest Nigeria - A mixed method study. Springer Science and Business Media LLC. 2024. doi: 10.21203/rs.3.rs-5451843/v1

[22] Ssensamba JT, Mukuru M, Nakafeero M, Ssenyonga R, Kiwanuka SN. Health systems readiness to provide geriatric friendly care services in Uganda: a cross-sectional study. BMC Geriatr. 2019;19(1):256. doi: 10.1186/s12877-019-1272-2 31533635 PMC6749715

[23] El-Jardali F, Fadlallah R, Daouk A, Rizk R, Hemadi N, El Kebbi O, et al. Barriers and facilitators to implementation of essential health benefits package within primary health care settings in low-income and middle-income countries: A systematic review. Int J Health Plann Manage. 2019;34(1):15–41. doi: 10.1002/hpm.2625 30132987

[24] Zimba CC, Akiba CF, Matewere M, Thom A, Udedi M, Masiye JK, et al. Facilitators, barriers and potential solutions to the integration of depression and non-communicable diseases (NCDs) care in Malawi: a qualitative study with service providers. Int J Ment Health Syst. 2021;15(1):59. doi: 10.1186/s13033-021-00480-0 34116699 PMC8196431

[25] Ogunyemi AO, Balogun MR, Ojo AE, Welch SB, Onasanya OO, Yesufu VO, et al. Barriers and facilitators to the delivery of age-friendly health services in Primary Health Care centres in southwest, Nigeria: A qualitative study. PLoS One. 2024;19(3):e0288574. doi: 10.1371/journal.pone.0288574 38502650 PMC10950227

