# Supplementary material for: Facilitators and barriers of general health service readiness in the primary level public health facilities of Baitadi, Nepal: A qualitative study
Source: PLOS Glob Public Health. 2026 Feb 23;6(2):e0006043. doi: 10.1371/journal.pgph.0006043 (PMC12928467; doi:10.1371/journal.pgph.0006043)
Supplement: S1 Data — (DOCX) [file pgph.0006043.s002.docx]

**Translated File**

**KII_A**

**Interviewer:** Namaste, Sir!
**Respondent:** Namaste!

**Interviewer:** As part of our study, we previously assessed the readiness of institutions, and this Community Health Unit was found to have a lower readiness score compared to others. Based on that, we are trying to explore the reasons behind this low score. To begin with, could you share your role, position at this health facility, and your experience working in the government health sector?

**Respondent:** First of all, I would like to express my gratitude for giving me the opportunity to share my thoughts. I’ve been working in the health sector for about 5-6 years, and it’s been around two and a half years since I joined this Community Health Unit. Here, I serve as a Sr. AHW and my main responsibility is to provide services to service users using the available resources. If resources are unavailable, we coordinate with the rural municipality or the health section to ensure the supply of necessary medicines and deliver services effectively.

**Interviewer:** Earlier, I mentioned that this unit has scored lower on readiness to provide quality services. In your opinion, what could be the reasons for this? What are the barriers?

**Respondent:** The main issue here is accessibility. The road to this area is challenging, making transportation of supplies difficult. Medicines are not directly supplied to this facility from the municipality; they go through Gokuleshwor Health Post, which then distributes them to multiple facilities from there. For instance, if 500 tablets arrive, they need to be divided among three facilities, leaving us with insufficient stock to meet the needs of the large number of clients here.

**Interviewer:** How has the rural municipality supported this unit?

**Respondent:** The municipality has provided significant support. For example, they allocated a budget of around NPR 300,000-400,000, which we used for renovations. Previously, this was a level-0 facility, but improvements have been made, such as electricity installation and repairing broken walls. Despite this, the level of support and coordination could be improved further.

**Interviewer:** You mentioned transportation challenges and support from the municipality. Despite this, what other factors might be contributing to the low readiness here?

**Respondent:** One issue is that this Community Health Unit is entitled to only 38 types of medicines, which are insufficient for the large number of clients we serve. Medicines are supplied to only health post in a ward. These medicines have to be distributed across three institutions. The number of service users has increased, but the supply of medicines has decreased. For example, in the Dilsaini rural municipality, there is a plan to purchase medicines worth 1.6 million. However, 1.6 million won't be enough to supply medicines to all health institutions here. If some medicines were purchased from the development budget and distributed to each health institution, it would have helped increase readiness.

We also don't have an account in our name to manage our own operations. Many things could have been managed better if the institution had its own account to handle administration. For instance, last year we had 25 deliveries. When the amount for 25 deliveries came, we had to go through the Gokuleshwar health post for the work. According to the management committee's decision, it would have been better if we could have procured medicines directly. Many things could have been managed that way, but due to this, readiness decreased.

**Interviewer:** Have any NGOs or INGOs supported this health unit?

**Respondent:** So far, no major support has been received from NGOs or INGOs, except for occasional assistance from the Family Planning Association, such as providing implants and other family planning services.

**Interviewer:** Do you think that if NGOs or INGOs provided support, the readiness of this unit would improve?

**Respondent:** Definitely! For instance, we urgently need a proper building with sufficient rooms and ventilation, a delivery room with proper roofing, and equipment like refrigerators. Support for such infrastructure would greatly enhance our readiness.

**Interviewer:** How is the coordination between the health unit and the health department? Could poor coordination be affecting readiness?

**Respondent:** When we submit request forms for supplies, the response is often delayed or inadequate. They tell us that they will give us a budget of one lakh, and that we need to complete certain tasks, but they don’t do in action. When we inform them, they say that medicines have already been sent, but they don't arrive. If the municipality ensured timely responses and supplies, our readiness would improve significantly.

**Interviewer:** What role does community engagement play in enhancing readiness?

**Respondent:** Community support is strong. They want to see this facility upgraded. We frequently engage with mothers’ groups to discuss health indicators and improve service accessibility. However, community contributions, such as providing land for infrastructure development, would help address some gaps.

**Interviewer:** How satisfied are the service recipients, and what feedback mechanisms are in place?

**Respondent:** Clients generally trust us because we are local and provide round-the-clock services when needed. However, we lack a formal suggestion box or system. Most feedback is provided verbally during interactions, and the main suggestion is to improve the availability of medicines.

**Interviewer:** Does being a local health worker help in trust-building?

**Respondent:** Absolutely. Being local allows us to provide services beyond regular hours, which builds trust. However, the unavailability of medicines sometimes affects client satisfaction.

**Interviewer:** How does supportive supervision impact readiness?

**Respondent:** Supervision is crucial. Periodic visits by the municipality or district health team to identify gaps and provide guidance would help improve our readiness. Some things need to be recorded in the register, and we have new staff as well. New staff don’t know everything yet. If someone from the municipality or district came occasionally to supervise us, review our mistakes, and provided advice like, "This is where the mistake is, and this is what you need to do," then our readiness could improve further.

It’s not that we’re unwilling to do things; we are already working on it. However, in some areas, you might say, “This is something you should do, this is your shortcoming, and you should address it.” But supervision is supervision, sir. If the health team from the municipality came to our health facility, reviewed our shortcomings, examined every register, assessed our management, and gave us advice and guidance, it would be much more effective

**Interviewer:** How does being in a rural area impact the readiness compared to urban health facilities?

**Respondent:** Rural locations face more challenges, such as delayed medicine supplies and infrequent monitoring. Urban facilities receive more attention and support, which is lacking here. This facility is located far from the district headquarters, and there are delays in transporting medicines to rural areas. Even during monitoring, visits are often made to health facilities in nearby urban areas. Advice and suggestions are mostly given to those nearby facilities, while such areas like ours are visited very rarely.

It seems like our readiness is low, partly because our complaints and the request forms we have submitted may not have been addressed. After we fill out request forms, it seems there is no consideration for the need in this area or a mindset that these requirements must be fulfilled. Negligence is the main reason, I would say. The supplies we request must be provided—that is my point. Our concerns also need to be heard. Once we report the problems we are facing, there should be action taken to address them.

**Interviewer:** There are different types of health facilities. Do you think readiness varies according to the type of facility?

**Respondent:** Of course, it differs, sir. Health posts are well-equipped with various services and facilities. In many health facilities, staff meetings are held where they discuss what is lacking, and they can fill out request forms to procure those things. There are also more staff in such facilities, and they have designated positions.

In contrast, community facilities don’t have designated positions. Staffing is done by the municipality. The concept is that since it’s a community facility, it probably won’t have adequate medicines or services. There are fewer staff, and medicines are not directly supplied from the municipality; they come through health posts.

**Interviewer:** How do training opportunities affect readiness?

**Respondent:** In our Dilasaini Rural Municipality, the positions seem to be fulfilled so far. However, when there is a training program, it is limited to two participants from the municipality. We receive no more than one or two training sessions in a year.

We haven’t received even one training session this year. If staff members who haven’t had the chance to participate in training could be included, it would make improving readiness much easier. It’s not sufficient for just one person to receive training; everyone should be provided training as per their needs.

**Interviewer:** What are the major challenges related to resources and supply?

**Respondent:** The municipality is well aware that the Community Health Unit in Pujarigaun has the highest number of service users and institutional deliveries. Despite knowing this, the supply of medicines here is insufficient.

We do not have account operations either. With 25 deliveries, some of the funds generated from our institution could be used to purchase medicines. This could help improve readiness to some extent.

**Interviewer:** How does leadership impact readiness?

**Respondent:** There needs to be leadership that listens to and addresses the concerns of the people and health institution staff. For instance, if we say, "Sir, we need this item, this medicine is unavailable, please ensure its supply, or we lack this equipment, and you need to provide it," there should be leadership to respond to such issues. The lack of response to these matters has also contributed to our reduced readiness.

While every in-charge voices their concerns, the municipality may also have its challenges. However, just as medicines are transported to other health institutions, medicines for the Pujarigaun Community Health Unit should also be delivered directly from the municipality, not via the health post. This would ensure timely delivery.

**Interviewer:** What are the challenges related to health information systems?

**Respondent:** There is a lack of internet for communication. You also experienced the problem when you came here and used the internet. Every health institution should have internet access, but we don't have it connected yet. However, our tower is under construction, and the internet will likely be operational in a few days. When medicines are running out, we inform through a notice one or two days in advance, saying "This medicine will run out soon." The lack of internet has caused a shortage in some areas as well.

**Interviewer:** How does staff motivation impact readiness?

**Respondent:** Staff motivation is critical. Recognizing and addressing their concerns can boost their confidence and enhance readiness.

**Interviewer:** Do cultural factors impact readiness?

**Respondent:** There isn't much of that, sir. Our cultural issues are more related to untouchability. Sometimes there are specific issues during childbirth. However, it’s not that everyone behaves in that way. It cannot be said that untouchability or cultural issues have reduced our readiness. Some aspects might have affected our readiness due to our own reasons

**Interviewer:** What would you suggest as the best ways to address the challenges and improve readiness?

**Respondent:** Timely supply of requested resources by the municipality and addressing minor gaps like setting up a suggestion box can make a big difference. While we are ready to handle smaller tasks ourselves, larger support is needed from the municipality. Proper coordination, planning, and action can significantly enhance the readiness of this facility.

**Interviewer:** Do you think something needs to be done at the policy level, or should the focus be on strengthening the implementation of existing policies?

**Respondent:** Delays in medicine procurement are major reasons. Whether this is due to political reasons or staff inefficiency, we are not sure. Our responsibility is to communicate with the concerned branch. For example, we notify the relevant staff, saying, "Sir, we need this medicine." We do not take these issues to political figures. Municipal staff have conducted monitoring visits and pointed out what is lacking. However, it’s not enough to just point out issues; they also need to take action and address them.

**Interviewer:** Finally, is there anything else you would like to share that I may have missed asking?

**Respondent:** In fact, I appreciate that you came here and took the time to understand the challenges of this place. Please convey the issues we face here to the health section such as, "These are the problems, and they are affecting the facility's readiness." On our part, we will do whatever is within our capacity to improve. However, our readiness is still low. For example, we need three dustbins, but we don’t even have one. We are currently using an empty bucket instead. Addressing such issues will help us enhance our readiness.

**Interviewer:** Alright, thank you for your time, sir.

**Respondent:** Thank you!

**KII_B**

**Interviewer**: Namaste sir
**Respondent**: Namaste

**Interviewer**: We previously did a quantitative assessment to evaluate the readiness of this health facility, and it was found to have lower readiness compared to other centers. Today, we want to discuss the barriers that contribute to this lower readiness. To start, could you please tell us about your role and experience here?

**Respondent**: Thank you. I have been working here for about eight years. I am currently serving as a Public Health Inspector and also holding the responsibility of the office head of the institution. For any health institution, readiness is essential to provide services to the clients. First, we need the infrastructure, human resources, and logistics, all of which should meet the government's standards or the requirements of the community.

**Interviewer**: What are the barriers to this readiness? What factors contribute to the low readiness? What could be improved to enhance readiness?

**Respondent**: When talking about barriers to readiness, there are three main factors: Physical infrastructure is significantly impacted by various issues. The increasing population and the high demand for services in this institution are factors at play. Additionally, Kesarpur Primary Health Center is now functioning as a referral center for three municipalities. A lack of sufficient physical infrastructure and facilities that are not easily accessible are barriers. If there is a shortage of human resources, that also becomes a barrier. The key issue is logistics; timely supplies and deliveries should be made within 12 months. If logistics are lacking, they also serve as a barrier. There is a noticeable lack of preparedness in the health institution. If these issues are addressed, we could easily be prepared.

**Interviewer**: You mentioned logistics supply. How is the logistics supply here, and how does the lack of medicine affect readiness?

**Respondent**: According to Nepal's new federal structure, this institution falls under the local government, and the municipality here is responsible for managing everything. In terms of logistics, the municipality opens tenders to supply logistics items. Sometimes, however, there is a shortage of staff within the municipality. The local government sometimes delays opening tenders or delivering supplies, leading to significant gaps. We request the local government to streamline this process and ensure smooth supply management so that there is no shortage of goods at any time throughout the year.

**Interviewer**: Various NGOs/INGOs support health facilities with infrastructure or technical support. Does Kesarpur Primary Health Care Center receive any such support, and how does it affect readiness?

**Respondent**: Yes, recently an organization called NEEDS provided plumbing for our water supply, and UNFPA contributed freezers for vaccine storage. Many organizations have directly or indirectly supported us to make the facility more effective and efficient.

**Interviewer**: The support from these organizations helps to increase readiness!
**Respondent**: Absolutely.

**Interviewer**: Now, with the federal system, the coordination among various agencies is important for supply management. As an executive here, what challenges have you faced in coordination, and how do they affect readiness?

**Respondent**: Indeed, in order to keep any institution fully prepared and ensure smooth service delivery, coordination is absolutely essential. The chairman of our health institution management committee leads our meetings. Through him, we communicate with the local government or relevant authorities, addressing issues and demands, and also directly request the municipality or local government through the health sector. Sometimes, however, there are shortages of staff and various procedural delays within the municipality, which can lead to delays in coordination.

**Interviewer**: Have you faced difficulties in coordination during your time in the executive position?
**Respondent**: Our health institution receives approximately 13,000 to 15,000 patients annually. This is the second busiest health facility in the district, after the district hospital in Baitadi. We now face a significant need for logistics compared to other health institutions. There are times when essential medicines and other logistics required for patient care run out of stock. This leads to situations where we have to listen to complaints from service users as well.

**Interviewer**: You've mentioned patient complaints. How engaged are the patients in providing feedback? What kinds of suggestions do they provide, and how do these help improve readiness?
**Respondent**: This region is remote, and the demand from patients is high. They often expect services like x-rays or ultrasounds to be available here. When these services are not available, we try to manage their expectations, but if all the necessary infrastructure, human resources, and logistics are available, it would be much easier to provide these services.

**Interviewer**: You have a suggestion box for feedback. What other mechanisms do you have for receiving feedback, and what challenges do you face in implementing them?
**Respondent**: We have a suggestion box, and patients also directly provide feedback. We conduct an annual social audit and public hearings where various suggestions and feedback are received.

**Interviewer**: Do negative feedback and dissatisfaction from patients affect readiness?

**Respondent**: We do not face much negative feedback, but we try to address all the suggestions and manage them effectively to improve the services.

**Interviewer**: Supervision is also important for service improvement. You mentioned that there has been no supervision recently. Do you feel that a lack of supervision affects readiness?
**Respondent**: Absolutely. There is often a lack of initiative among some staff members, and sometimes it feels like there's little sense of urgency. This health institution is closest to the local government office, yet we have not received sufficient supervision. If higher authorities were to come for regular supervisory visits and provide supportive supervision on logistics, human resources, and staff matters, it would boost the morale of the staff. This would increase readiness and, in turn, directly benefit the service users.

**Interviewer**: The location of the health institution also plays a role. Baitadi has two primary health centers: one in Patan and one here. Patan is closer to the highway. Do you think the geographical location affects readiness?

**Respondent**: Our center is located at a very accessible place, closer to the road than Patan. We receive patients from three municipalities, so we need to be well-prepared. Previously, when accidents occurred nearby, we were able to manage the situation effectively.

**Interviewer**: Does the geographical location impact logistics supply?
**Respondent**: There are no significant challenges with logistics supply now because we are near the road, so geographical difficulties do not impact the supplies.

**Interviewer**: Different types of health facilities, like health posts, primary health centers, and health units, may have different readiness levels. What do you think about this?
**Respondent**: Health posts are generally at the periphery, but here, the patient load is higher, so we need to be more prepared. We offer 24-hour services, which means we must be more prepared than other facilities.

**Interviewer**: How are refresher trainings for health workers? What challenges do you face in organizing various training programs?

**Respondent**: The government organizes training programs, but refresher training is somewhat lacking. Training programs for new staff are organized periodically.
**Interviewer**: You mentioned medical supplies earlier. What barriers do you face in obtaining medical supplies?

**Respondent**: We request supplies through the local government. The procurement process is slow, and sometimes there are delays in delivery. The time it takes to complete the procurement process can lead to difficulties in supply. The local government needs to find ways to make this process smoother.

**Interviewer**: Leadership, whether at the primary health center, health department, or municipality level, plays a role in improving readiness. How do you think leadership affects readiness, and what barriers arise if leadership is weak?

**Respondent**: In the federal system, the local level has administrative heads. If leadership recognizes the importance of health services, as seen during the COVID-19 pandemic, readiness improves. Effective leadership ensures that health services are prioritized, which leads to better preparedness.
**Interviewer**: How is the health information system and reporting at this facility? What challenges do you face?

**Respondent**: There are no major difficulties with the health information system.

**Interviewer**: How does good morale and motivation among staff help improve readiness?
**Respondent**: Supportive cooperation and coordination from all staff are crucial for managing the health facility well. When everyone is motivated to ensure the facility is well-managed, it improves readiness, and services become more efficient.

**Interviewer**: Are there cultural practices in this region that present challenges to improving readiness?
**Respondent**: There are some traditional practices in the region, but they have been significantly reduced over time. These practices don't directly affect the preparedness of the health facility. However, they might affect overall health indicators.

**Interviewer**: What do you think is the best practice for improving readiness?
**Respondent**: The best practice is having the willpower to ensure the institution is well-managed. Monitoring, coordination with health authorities, and clear communication can greatly improve preparedness.

**Interviewer**: Do you think current policies are sufficient to improve readiness, or should there be changes?
**Respondent**: If policies are adjusted to make the procurement process faster and smoother, it would significantly help improve readiness.

**Interviewer**: What kind of policy changes would you recommend?

**Respondent**: Policies should allow for quicker procurement processes, especially for essential items like medicines, without lengthy tendering procedures. There should be alternative mechanisms in place for quick supply to improve readiness.

**Interviewer**: Thank you, Sir! If you have any final comments on readiness, please share them.
**Respondent**: Thank you! Well-managed services require good preparation. Without the necessary resources, even soldiers cannot fight. We need to be prepared to provide the best services to our community.

**Interviewer**: Thank you!
**Respondent**: Thank you!

KII_C

**Interviewer:** Sir, Namaste! Last time, we conducted a quantitative assessment for the readiness of health facilities. In that assessment, this health post was found to have lower readiness compared to others. Today, we would like to discuss the reasons behind this lower readiness and the barriers associated with it. To begin, could you please share your experience in the public sector and the position you currently hold?

**Respondent:** It has been almost 26 years of service, sir. Initially, I worked in the old Dasharathchand, then in Giregada, followed by Sigash, and then Sittad of Dogadakedar. Now, I have been at Thaligada Health Post in Dasharathchand Municipality for about five years. My position is Sr. AHW Officer.

**Interviewer:** As we discussed earlier, the readiness of this health facility is lower. What do you think are the reasons for this low readiness?

**Respondent:** Firstly, there is no building of our own. This building doesn’t belong to us; it belongs to an organization called Samaj Sudhar (Society Improvement). Without our own building, it is difficult to manage many things. For example, we need to dig pits, like for placenta, and we need to construct them. Moreover, there is no proper road connection. Dasharathchand Municipality has hired staff compared to other municipalities, but there is still a lack of necessary equipment and other essentials. That’s why we are behind in terms of readiness.

**Interviewer:** NGOs and INGOs often provide support to improve the readiness of health facilities. Has this health post received any assistance from any non-governmental organizations?

**Respondent:** This is the oldest municipality, but NGOs and INGOs don’t work here. They usually support rural municipalities or the old VDCs (Village Development Committees). There has been no support here.

**Interviewer:** If there were support from NGOs/INGOs, how could the readiness be improved?

**Respondent:** We didn’t even have beds for patients here. Just a few days ago, the municipality provided them. There is a lack of rooms, so we are managing by creating partitions. Recently, the ward allocated some funds, but we don’t even have curtains. We received a low score because we lack basic items like pillows under the minimum service standards. We have no financial resources of our own. Last year, the municipality gave NPR 11,000 as administrative expenses, and the ward provided NPR 40,000. This was spent on partitions and repairing a broken toilet door. If additional funds were provided, gradual improvements could be made.

Before that, the municipality didn’t even allocate administrative expenses. They sent two packs of pens and one pack of photocopy paper, and that was it. For other things like soap and phenyl, we have been managing them ourselves. This year’s funds are supposed to arrive, but we haven’t received them yet. For now, we are managing things on our own.

**Interviewer:** Coordination is also necessary for readiness, whether with the municipality or the ward. What challenges are faced during coordination that affect readiness?

**Respondent:** We hold monthly meetings, sir. We discuss issues during the meetings, and we say what’s lacking. They respond by saying, “It will happen next time,” but it doesn’t. There are three facilities under Ward 6: Shera Municipality Center, Titara Municipality Center, and this one. As funds need to be distributed among all three, the budget we receive is smaller. If there were only one facility, we would have received NPR 1.5 lakhs, but now we only receive NPR 40,000. The difference between NPR 40,000 and NPR 1.5 lakhs is significant.

**Interviewer:** What kind of complaints or suggestions do you receive from service seekers?

**Respondent:** There are no complaints, sir. Whether it’s limited space or a lack of equipment, we utilize our experience to provide the services we can. We have been providing services whenever needed. Even after 4 PM, if someone comes, we open the facility and offer services.

**Interviewer:** What mechanisms are in place to gather suggestions from them?

**Respondent:** We have a suggestion box, but no one uses it. People don’t verbally express their suggestions either. The box is kept there just in case someone uses it, but it’s mostly unused.

**Interviewer:** Supervision is also important. How often does supervision happen, and how does inadequate supervision affect readiness?

**Respondent:** Supervision happens very rarely, sir. Just yesterday, after about six months, a municipal team came to evaluate the Minimum Service Standards (MSS). Despite years of work, there isn’t much flexibility to act independently due to financial constraints and current regulations. Health offices say, “You need to do this,” but they don’t offer any support. Political leadership at the municipality says they’ll handle things, but by the time their term ends or halfway through another term, there’s little progress. However, under good governance, some improvements have been made, like the introduction of electronic attendance and internet facilities. Online data entry wasn’t possible before, but now it is. Gradual improvements are expected, but it’s slow.

**Interviewer:** How does supervision affect readiness?

**Respondent:** Supervision has a positive impact, sir. It helps identify shortcomings we might have missed. Suggestions like “This should be done differently” help improve the situation. It definitely makes a difference.

**Interviewer:** Are there any geographical barriers affecting readiness?

**Respondent:** The health post doesn’t have road access. Transporting supplies is challenging. Even though the road is nearby, it costs at least NPR 500 to bring materials here. Additionally, the land doesn’t belong to us. However, a donor has proposed donating land near the road, and we’re moving forward with that process. Hopefully, it will be completed in a couple of months.

**Interviewer:** How often do health workers receive training, and how does it affect readiness?

**Respondent:** Training helps with areas we’re unfamiliar with. Trainings are conducted, but there’s a difference between the old and new trainings. Previously, expert trainers from outside used to come, but now trainers from within the district conduct them. Sometimes I provide, and other times someone else does. The quality of the training isn’t as it used to be, and it feels inadequate.

**Interviewer:** What challenges cause delays in the timely supply of medicines?

**Respondent:** Delays happen because the municipality doesn’t issue tenders on time. They wait for a convenient time or budget adjustments, which delays the process. For instance, this year, they could have tendered in Shrawan (July-August) but did it only in Kartik (October-November). Additionally, the company provided medicines into three lots, which compromises the quality of medicines and further delays their supply.

**Interviewer:** How does the leadership of the municipality, ward, or health facility impact readiness?

**Respondent:** Leaders at all levels understand health issues but don’t act promptly. Budget constraints or busy schedules often come up as excuses. In some places, proximity between the ward and health facility makes it easier to work together. However, in our case, the ward office is far away. Leadership knows everything, but ultimately, it all comes down to budget limitations.

**Interviewer:** Are there any challenges in health information management?

**Respondent:** Not many, but there was a slight issue with LMIS recently. A one-day refresher training addressed it. Sometimes there are system errors, like data not displaying due to heavy loads or poor internet connectivity.

**Interviewer:** How does having motivated staff contribute to readiness?

**Respondent:** Cooperation among staff is there, and motivation isn’t an issue. However, the lack of infrastructure, such as buildings, is a major barrier. For example, we need a separate room for autoclaving as per MSS, but we don’t have space. Even temporary solutions like tin sheds aren’t feasible. Despite motivated and experienced staff, the lack of infrastructure hampers progress.

**Interviewer:** Do any cultural practices in the community impact readiness?

**Respondent:** Not really, sir. There are traditional practices, but people are open to advice and suggestions. It’s not a significant challenge.

**Interviewer:** What needs to be done to improve readiness, apart from infrastructure?

**Respondent:** A building would solve many issues. With a proper building, we can have separate spaces for OPD, safe motherhood services, family planning, and medicine distribution, which would improve our scores. Road access would make things easier too. Once the building is in place, we can focus on equipping it.

**Interviewer:** Do you think policy changes are needed to improve readiness, or should the existing policies be better implemented?

**Respondent:** The current MSS standards seem mismatched, sir. The same standards apply to district hospitals, provincial hospitals, and health posts, which isn’t practical. Health posts need separate MSS standards. For example, our health post achieved 68% under MSS. If there were separate standards, we might have reached 80-85%. Adjusting these standards could bring improvement.

**Interviewer:** Finally, is there anything I missed regarding barriers or anything you’d like to share?

**Respondent:** Nothing specific, sir. However, constitutional amendments are being discussed. Health services should ideally remain under a central integrated system rather than being decentralized. Under the current local system, political influence from ward chairs and others creates issues. If health services were managed centrally, many problems could be solved.

**Interviewer:** Thank you, sir.

**KII_D**

**Interviewer:** Namaste Sir! Our assessment of readiness across various municipal health centers shows that this center has a lower score compared to others. We would like to discuss the reasons for this. Can you first share a little about your experience and role in the public health sector?

**Respondent:** I have been working here since 2074 (2018). Before this, I worked at a health post and a birthing center. In terms of experience, sir, people don't come here much, like in the urban areas. It's a small, basic healthcare setup. I have provided all the services I can. If there’s something more severe, we refer the patient elsewhere. Here, it's just basic care.

**Interviewer:** Compared to other municipal health centers, Nwala Urban Health Center’s readiness seems lower. What do you think are the reasons or barriers for this?

**Respondent:** One reason could be that the building is new. We still need to put up suggestion boxes, signboards, and a citizen charter. We haven't received any medicines since the month of July. That's why there is a shortage of medicines. When we talk to the municipality about it, they say that medicines come late because of delays in the procurement process process. The medicines that were supposed to arrive by the end of June haven’t come yet, and even the new ones that were expected are uncertain.

**Interviewer:** Do you think the long tender process is the reason why medicines aren't arriving on time?

**Respondent:** Yes, that’s what I think. That's also what we are told. When we bring up the issue, they say that medicines aren’t available because of delays in the tender process.

**Interviewer:** Some health institutions receive support from NGOs or INGOs. Is there any such support at this center?

**Respondent:** Currently, there is no support from any organization.

**Interviewer:** If an organization were to provide support, how do you think it could help in removing the barriers?

**Respondent:** If an organization provides support, the biggest help would be if they could supply the medicines we’ve requested. That would solve most of our issues.

**Interviewer:** While working at the grassroots level, you must have had to coordinate with various agencies. What challenges have you faced during coordination, and how has this affected the center’s readiness?

**Respondent:** One problem is the network connectivity. At times, we can’t even make a call when needed. The network works a bit when there is sunny day, but once the sun sets, it goes off. Even on cloudy days, there’s no network. So, updating ELMIS becomes impossible when needed. We have to go to the municipality once a month just to update the information. That’s why we don’t even know how much medicine is available or not. The medicine requests are delayed because of the tendering issues.

**Interviewer:** Has there been any feedback or complaints from the community regarding the shortage of medicines?

**Respondent:** Not really, sir. The common issues here are headaches, toothaches, and stomachaches. Even with just paracetamol, we can manage most of them. Some people may need more, but we haven’t received any complaints. Maybe if we had suggestion boxes, we would get some feedback, but we haven’t set those up yet. For more serious patients, they go to places like Dhangadhi or Dadeldhura for treatment.

**Interviewer:** Supervision is important for increasing readiness. You mentioned that no one has come for supervision in the last four months. Do you think the lack of supervision has contributed to a decline in readiness?

**Respondent:** Yes, supervision is essential. It makes a huge difference when someone visits in person to assess the situation compared to just being told over the phone. It would help if someone came at least once every 2-3 months to directly observe the situation.

**Interviewer:** Do you think the geographic remoteness of the area has also contributed to the low readiness?

**Respondent:** Yes, that’s true. The geographical remoteness is a big challenge. The road was only cleared recently, and even when monitoring visits are scheduled, the vehicle can only reach a certain point, and the rest has to be walked. This causes delays in supply and monitoring. If there was better transportation and communication, it would be easier to meet the readiness requirements.

**Interviewer:** Do you think the type of health institution affects its readiness?

**Respondent:** In my experience, the type of health institution doesn’t make much difference in readiness. Whether it’s a health post or a municipal health center, the medicine supply should be the same. But because of geographic remoteness and network issues, we face delays here.

**Interviewer:** How does the lack of adequate training for health staff affect readiness?

**Respondent:** Refresher training is essential. If staff haven’t received training for a few years, they might become a bit confused. Refresher training would make things easier. The local government should arrange it if there’s a budget. However, the municipality hasn’t organized such programs so far, citing budget issues.

**Interviewer:** How do you think good leadership could impact readiness at the health center?

**Respondent:** If the leadership understands the issues and knows how to solve them, it would make it easier to improve readiness. Local government leadership needs to be aware and knowledgeable to address the challenges effectively.

**Interviewer:** What barriers exist in relation to the health information system?

**Respondent:** There are no issues with paper-based recording and reporting, but we face problems with the online system because the internet doesn’t work here. We have to go elsewhere to work on the system. There are issues with both the ELMIS and HMIS systems due to the lack of internet access.

**Interviewer:** Do you think that staff motivation, both monetary and non-monetary, could help improve readiness?

**Respondent:** Monthly meetings to identify problems and discuss solutions could help. If staff know each other’s issues and resolve them together, readiness will improve.

**Interviewer:** What efforts have you made to increase readiness while working here?

**Respondent:** The main issue is geographical remoteness. If there was consistent transport and communication infrastructure, it would be much easier to manage. The transport needs to be available year-round, and the communication network should work 24/7. That would make everything much smoother.

**Interviewer:** You mentioned that the tender process is lengthy. What could be done to ensure medicines are supplied on time?

**Respondent:** The municipality should shorten the tendering time and only award contracts to those who can deliver on time. If suppliers delay, there should be legal consequences as per the policy.

**Interviewer:** What policy changes do you think could help improve readiness?

**Respondent:** There should be a policy that ensures medicines are delivered on time, with legal consequences for suppliers who fail to meet deadlines. Readiness depends not only on medicines but also on transportation, communication, and electricity. If these services are ensured and laws are strictly enforced, readiness will improve.

**Interviewer:** Finally, do you have anything else to share related to readiness?

**Respondent:** Regular staff meetings will help improve readiness. If supervision happens every 2-3 months, it will allow the authorities to directly assess the issues and solve them. The local government should ensure transportation and communication facilities work 24/7.

**Interviewer:** Thank you for your time!

**Respondent:** Thank you!

KII_E

**Interviewer:** Namaste Sir, today we will discuss why the readiness of this health institution is low, what barriers exist. To start, how many years have you worked in the government sector? Please share your experience and role at this health institution.

**Respondent:** I have been working in the government sector for almost 10 years, sir. Before that, I also worked on a contract basis for 3/4 years. From my experience, there are certain geographical challenges, conservative mindsets, and a lack of public awareness in the community. To solve this, we need to coordinate and work with the public, ward chairman, and municipal representatives.

**Interviewer:** We will discuss coordination later, sir. Now, among the other basic health service centers in the district, this institution has the lowest readiness. What do you think is the reason for the low readiness? What barriers are there?

**Respondent:** In the past, as we moved towards federalism, some municipalities were formed, some rural municipalities were formed, and in some wards, there were registered health institutions, but in some wards, there were none. In the places where there were no institutions, we provided services through the Basic Health Service Center. Now, due to a lack of resources, geographical infrastructure, and insufficient funds for building the required facilities, it has been difficult to provide services. For everything to be efficient, all the infrastructure and services must be available.

**Interviewer:** NGOs/INGOs also help increase the readiness of health institutions. Has any NGO/INGO supported this health institution?

**Respondent:** This institution has not yet been registered, and since it was developed from the Health Desk into a Basic Health Center and has been providing services, we have not had the opportunity for any support or coordination from an NGO/INGO. If any institution offers help, we are open to accepting it.

**Interviewer:** How do you think NGO/INGO support could help improve readiness?

**Respondent:** For the improve in data indicators, spreading public awareness, eliminating conservative practices, and helping to procure the required materials would improve readiness. For example, we don’t have our own health building here. If an NGO/INGO helped with constructing the building and provided necessary resources, it would greatly contribute to improving readiness.

**Interviewer:** Since federalism, we now have three levels of government. Local governments and other health institutions are also operating. When working here, you must coordinate with local government or other health institutions. What challenges exist in this coordination that are affecting readiness?

**Respondent:** In the past, due to the lack of a health institution in this ward, we had to provide services from the Health Institution of next ward. Since last July, all health institutions, health section, and municipalities have coordinated to provide health services from the respective wards.

**Interviewer:** Is it because the health institution has only been operational since last July that readiness is low?

**Respondent:** Yes. We are still in the process of securing resources. We are trying to gather all necessary equipment, request human resources, and add services to increase readiness.

**Interviewer:** Have there been any complaints about a lack of medicines here?

**Respondent:** Earlier, it was a Health Desk, and we didn’t even have the medicines that we currently have. Before, people had to go to another ward to receive services. Since July, after the municipality started sending medicine here, the people here are happy with the services. They don’t have any complaints, and we are in the process of requesting medicines based on consumption.

**Interviewer:** Supervision is also important for increasing readiness. You mentioned that there hasn’t been any supervision in the last four months. Could the lack of supervision be a reason for the low readiness?

**Respondent:** The health workers from the municipality oversee us. We make phone calls, and there are indirect programs and monthly meetings. There is a process for visits from the ward as well. Since this is a new institution, we are focused on gathering resources and providing services. The ward chairperson and municipality are positive. Our common goal is to gather resources and improve services.

**Interviewer:** While there may be calls and monthly meetings, has anyone come specifically to assess how services are being provided, check if there are medicines, and offer suggestions?

**Respondent:** The health section officials visited during the inauguration. The municipality tracks the supply of medicines and their expiry dates online. Other issues are regularly discussed in the monthly meetings. We review all health institutions during these meetings.

**Interviewer:** Despite this, why is there still a shortage of medicines here?

**Respondent:** We request medicines based on the patient load and severity. We order medicines based on what we can use. Since this is a new institution and there hasn't been a significant patient load, we only request medicines after the patients arrive, as some, like metformin, might expire if ordered in advance. We only request medicines based on consumption. For example, if there are no TB patients, there is no need to request TB medicine.

**Interviewer:** Could the geographical remoteness be a factor affecting readiness?

**Respondent:** Yes, the geographical challenges affect readiness due to limited physical infrastructure and human resources. The transportation of goods is also difficult due to the distance. There is also a lack of public awareness in the community. These factors impact readiness.

**Interviewer:** Staff training is also crucial. How much training has been provided here, and how does the lack of training affect readiness?

**Respondent:** Trainings have been conducted, but it would be better if all staff members participated in the process. For example, there are malaria and mental health training programs. It would be good if everyone could attend compulsory training. If the same people are trained each year, it can be difficult to provide effective services. Each year, different people should be trained.

**Interviewer:** What challenges are there in transporting medicines and other supplies?

**Respondent:** We send request forms and have to carry the medicines ourselves. If it's difficult to transport the supplies, we even use tractors to bring them.

**Interviewer:** How does the leadership of the health institution or municipality affect readiness?

**Respondent:** Leadership is essential. It is important to identify where the deficiencies are. Everyone must take responsibility for their tasks. Without leadership, no work can be done. Leadership is the backbone of any work.

**Interviewer:** What issues exist with recording and reporting at this institution?

**Respondent:** Since this is a new institution, there are challenges. We don't have a laptop, but we have requested one at the municipal level. We also don’t have our own internet, but they are positive and say they will provide it. It takes time to complete the process. As a new institution, everything is gradually becoming available.

**Interviewer:** Motivation is also important for staff. How does motivation impact readiness?

**Respondent:** Motivation is necessary. We conduct staff meetings, divide tasks, and ensure that everyone takes responsibility with integrity. We must approach work as a duty.

**Interviewer:** What do you think should be done to increase the readiness of this institution?

**Respondent:** First of all, we need a building to increase readiness. The lack of space is the biggest issue. Problems with staff, infrastructure, and transporting goods are also challenges. If these issues are addressed, readiness will improve. However, it takes time to gather resources.

**Interviewer:** Finally, if you have anything to share regarding readiness, please feel free.

**Respondent:** I’ve worked in many places in the course of my career. I’ve also worked at the district level and had the opportunity to learn from doctors. I am very happy internally to be able to work at the community level and in the operation theater as well. However, it saddens me when I am unable to provide the services I want due to a lack of resources. It makes me feel inadequate. But as long as I live, I believe that service is a duty, and I will continue to work with responsibility to heal the wounds of the suffering.

**Interviewer:** Thank you for your time, Sir.

**KII_F**

**Interviewer:** Namaste sir!
**Respondent:** Namaste

**Interviewer:** Previously, we conducted a quantitative assessment of readiness. In that, this Primary Health Center (PHC) showed better readiness compared to another PHC in Baitadi. In this interview, we aim to discuss why your PHC achieved better readiness and the facilitators contributing to this. To start, what is your position here, and could you share your experience in the government sector?

**Respondent:** I am a Public Health Inspector. I have 19 years of experience in the government sector and have been working at Patan Primary Health Center for the past 5 years.

**Interviewer:** Why do you think your PHC has better readiness compared to other PHCs?

**Respondent:** Primarily, after federalization, Patan Municipality has been performing exceptionally well among the 10 municipalities in Baitadi. Within Sudurpaschim Province, Patan Municipality might even rank in the top 10. There has been notable progress in the health sector here, with dedicated efforts from all healthcare workers. Before federalization, Dr. Gunaraj Awasthi worked in Baitadi for about 8–9 years, leaving a significant impact with his vision for improvement. We have followed his approach since then. Regarding medicine, the municipality ensures a timely supply of required medicines.

**Interviewer:** In some health institutions, NGOs/INGOs support readiness improvement. Has any organization supported your PHC?

**Respondent:** NGOs haven't supported us much. However, German Med Nepal recently provided some lab equipment this year, including machines for thyroid and CBC testing.

**Interviewer:** How is the coordination between your health facility and the municipality regarding medicine and materials?

**Respondent:** It's very good. We have frequent discussions and coordination with municipal health section coordinator about medicines and materials. Recently, the government has added some medicines that we receive for free, particularly some heart-related medicines that were not available before. Previously, we used to provide these medicines through the health insurance program. I had coordinated with the health section about the need for their supply. Initially, the health section informed us they were unaware that the government had made these medicines free, and they had already initiated a tender process. However, later they coordinated with the district office to provide these medicines to us. Currently, the municipality has completed the tender process, so these medicines will now be supplied by the municipality. In any case, our coordination with the municipality regarding necessary materials and medicines is excellent.

**Interviewer:** How do you gather feedback from service users, and how does it help improve readiness?
**Respondent:** Patients from many places visit us, including from different municipalities in Baitadi and occasionally from Darchula due to the road proximity. In Baitadi, the health insurance program is implemented only in two places: Baitadi District Hospital and Patan Primary Health Center. As a result, many patients from various locations come to us. Due to limited resources, the primary health center cannot perform all types of diagnoses. While initial diagnoses are conducted elsewhere, patients often visit us for follow-ups and to obtain necessary medicines. We have also installed a suggestion box to collect feedback.

**Interviewer:** Nowadays, we emphasize supportive supervision, not just inspection. How frequently do you receive supervision from the municipality, health office, or higher levels, and how does it contribute to readiness?

**Respondent:** We receive regular supervision from the municipality and district office. If there is an issue with the cold chain, cleanliness, or materials, they supervise and guide us on how to address it. We implement the feedback provided through supervision and also request them to continue regular supervision as it helps us move forward. Supervision is also conducted by the Provincial Health Directorate.

**Interviewer:** Patan Municipality is located in an accessible area geographically. Does this geographical accessibility help in improving readiness?

**Respondent:** Definitely. Geographical and transportation accessibility have been beneficial. If we face a shortage of supplies, they can be delivered from even Kathmandu or Dhangadhi within a day or two, which is certainly advantageous.

**Interviewer:** How often do training programs take place for healthcare workers, and how do these contribute to improving readiness?

**Respondent:** The municipality identifies which staff members require what type of training and what training they have already received. Based on this, arrangements are made. Compared to other health institutions, staff at primary health centers receive more training opportunities, which has been helpful.

**Interviewer:** How does access to internet facilities and other conveniences contribute to readiness?
**Respondent:** It has definitely been beneficial. We have NT Fiber internet here, which is both affordable and fast. Previously, we used Everest's internet, which was neither fast nor cost-effective. We used to pay NPR 3,300 per month for Everest internet, but now with NT Fiber, we manage with NPR 1,000 per month. The good network makes Zoom meetings and other online activities easier.

**Interviewer:** If political or health leadership is sensitive to health issues, it make things easier. What kind of leadership have you experienced, and how has it contributed to readiness?
**Respondent:** After federalization, this is the second local election. In Baitadi, though other sectors have not seen much progress, there has been significant improvement in health. Patan Municipality has even received cash awards from the province for its achievements. The current representatives are also supportive of health sector improvements, continuing the good reputation Patan Municipality has built. The health section coordinator is very dedicated, and health workers within Patan Municipality are highly motivated and proactive. They are committed to maintaining the reputation the municipality has earned.

**Interviewer:** If staff are highly motivated, how does it contribute to readiness?
**Respondent:** We hold monthly meetings in all health institutions, and it has been decided that each institution should also conduct staff meetings and management committee meetings every month. During these meetings, we discuss weaknesses, how to improve them, and work accordingly. We regularly discuss training requirements for staff to make services more effective. This approach has increased staff motivation and improved readiness.

**Interviewer:** If management committee guidelines and a citizen charter are in place, what impact would that have on readiness?

**Respondent:** If there are guidelines, even if there is a leadership or member change, it becomes easier to operate the institution in a consistent manner as per the guidelines. People cannot act however they please. This ensures transparency, and the work is done more effectively, which supports readiness. Now, with the citizen charter, it becomes easier for service users. It also creates a certain level of transparency. It is said that every institution should have one. Rather from the perspective of a health institution, it is important for the service users

**Interviewer:** Among the many facilitators we discussed, what do you think is the best for improving readiness?

**Respondent:** Commitment is the most important. Punctuality, dedication to assigned duties, serving patients with a kind attitude, and addressing feedback are crucial. Everyone has weaknesses, but addressing them systematically is what matters. This has been our focus.

**Interviewer:** If the good practices implemented by this health institution are to be replicated in others, what would be your suggestion?
**Respondent:** The key is patient behavior. Treating them with utmost care is essential. We may not always have all the medicines available, so we coordinate with the health office and municipality for supplies. If that doesn’t work, we have a management committee fund to procure and provide medicines. This has been effective in ensuring a consistent supply of essential medicines. Over the past year, there have been no complaints about medicine shortages, largely due to the municipality's support. While we coordinate with the health office, it also requires constant effort and persistence to ensure services are provided.

**Interviewer:** Readiness in Baitadi district appears lower. What suggestions do you have to improve health readiness across the district?

**Respondent:** The key lies with the municipalities. They should learn from each other's good practices and move forward. The health office in Baitadi has been conducting meetings in various municipalities. For example, one meeting might be held in Patan Municipality, the next in Surnaya or Purchaudi, allowing for knowledge sharing. This feedback helps identify what medicines need to be supplied and other requirements. Not all medicines are needed everywhere; for example, certain medicines might not be required in Surnaya but are necessary in primary health centers with advanced services like X-ray facilities. Medicine supply should be based on the specific needs of each health institution, ensuring optimal utilization and minimizing wastage. Push systems often result in medicines expiring unused. Last year, atenolol 50 mg had to be discarded due to lack of demand. The health office should focus on demand-based supply.

**Interviewer:** Do you think policies need to change to improve readiness, or should there be more emphasis on implementing existing policies?

**Respondent:** Policies need to change. The staffing pattern is outdated, based on the 2048 B.S. (1991 A.D.) structure, which is insufficient. The physical infrastructure is also inadequate. This primary health center is in a dilapidated state, lacking space for various equipment. For example, we need a three-phase electrical line, which we've been trying to get for three years without success. Although efforts were made to establish hospitals in all municipalities during Prime Minister KP Sharma Oli’s tenure, progress has been slow. We are providing 24-hour services here with the existing staff, which is challenging. Staff work both day and night, which makes it difficult to operate smoothly without proper shifts. Therefore, changes are necessary.

**Interviewer:** Is there anything else you’d like to share regarding readiness that we haven’t covered?
**Respondent:** I believe everything important has already been discussed. There’s nothing more to add.

**Interviewer:** Thank you, sir.

**KII_G**

**Interviewer:** Namaste, sir.
**Respondent:** Namaste.
**Interviewer:** Previously, we assessed readiness, and this Basic Health Service Center, Takare, showed better readiness compared to others. In this interview, we want to discuss the reasons behind this improved readiness. To start, how long have you been working here, and what is your position?
**Respondent:** I’ve been working here for about three years, and I’m currently serving as a Health Assistant. The higher readiness here is mainly due to the good support provided by the municipality. All essential drugs supplied by the government are available. Supervision also occurs regularly, both from the municipality and the health office. When they visit, they come with a proper plan, which we follow to improve readiness. That might be why our readiness has improved.

**Interviewer:** In some health institutions, NGOs/INGOs also play a role in improving readiness. Have any organizations supported your facility in this regard?
**Respondent:** Yes, NGOs/INGOs have supported us. An NGO helped improve the delivery room. They installed tiles, built an attached toilet and bathroom, set up water taps, and installed a water tank. They have also supported us in infection prevention.

**Interviewer:** Coordination is also important. How has coordination contributed to improving readiness here?

**Respondent:** At the beginning of the fiscal year, we prepare an annual action plan. This plan includes details such as when management committee meetings and staff meetings will be held, which medicines are running low, and where to source medicines if unavailable at the municipality. We coordinate accordingly. The health section coordinator has been very supportive and ensures that any required medicines are provided when we inform him. The coordination has been very effective.

**Interviewer:** What kind of suggestions do you receive from the community, and how do you collect and address them?

**Respondent:** Some suggestions from patients are direct, while others are indirect. We have also set up a suggestion box to collect feedback. Additionally, we engage with the community through mothers’ group meetings and school health programs. We take their suggestions, coordinate with the management committee and municipality, and work towards resolving the issues.

**Interviewer:** Supervision is very important for improving readiness. Nowadays, we emphasize supportive supervision. How often does higher-level supervision occur here, and how does it help in improving readiness?

**Respondent:** Supervision is conducted every six months for the Minimum Service Standards (MSS). During this process, we review the six-month progress report. Indicators not meeting the standards are addressed either by staff, the management committee, or the municipality. For instance, indicators that staff can handle are managed internally, while others are resolved with the municipality's support. Since our health institution is located near the road, supervision also happens frequently. Officials from the district health office, municipality mayor, and health section coordinator visit us regularly. They provide guidance and occasionally organize health camps as part of their support.

**Interviewer:** How has the geographical advantage contributed to the readiness of this health facility?
**Respondent:** Since the facility is located near the road, it has been easier to bring in delivery cases and improve other indicators. Municipality officials visit frequently, and we can easily communicate our issues. Coordination has also been facilitated. Medicine supply is smooth as we can safely transport medicines at any time. Additionally, one staff member resides nearby, which makes it easy to receive medicines whenever they are sent. We also provide 24-hour services.

**Interviewer:** How does readiness vary based on the type of health facility?
**Respondent:** Ideally, readiness should be high in all health facilities, and there should be no low readiness. Indicators are generally similar across facilities. Once the building infrastructure is available, there should be no significant differences in other areas. Coordination with the municipality is key to managing medicine supply.

**Interviewer:** The physical infrastructure here is good. Has it contributed to improving readiness?
**Respondent:** Yes, the availability of physical infrastructure has significantly contributed to improving readiness. It plays a crucial role in managing other aspects of the facility.

**Interviewer:** How does training staff from time to time help improve readiness?
**Respondent:** In our health facility, we list the training required and note which staff members have received it and which have not. For those who haven’t received specific training, we coordinate with the municipality and the health section to request it. We ensure that untrained staff attend the training. Periodic refresher training is also necessary. Trained staff can address shortcomings and deliver better services, ultimately improving readiness.

**Interviewer:** How has support from the municipality or the leadership of the health section contributed to improving readiness?
**Respondent:** Speaking of leadership, we have a five-member team at the health facility. Any decisions we make in staff meetings are forwarded to the management committee and, from there, to the health section. The health section coordinator takes these matters to the executive committee. Following this process ensures good responses and support for our requirements.

**Interviewer:** How have staff meetings and management committee meetings contributed to improving readiness?
**Respondent:** In staff meetings, we discuss the standards required for service delivery. This includes recording, reporting, and reviewing indicators. We meet monthly and, if necessary, twice a month to discuss our progress and plan future actions. Issues discussed in staff meetings are forwarded to the management committee, which understands the priorities and plays a key role in improving readiness.

**Interviewer:** What kind of staff motivation have you received, and how has it helped improve readiness?
**Respondent:** We have received incentives, such as a reward of NPR 5,000 from the municipality for good work. The ward also recognizes one volunteer and one staff member annually for their excellent work. These initiatives have boosted staff morale and motivated us to perform better, contributing to improved readiness.

**Interviewer:** If management committee guidelines and a citizen charter are in place, what impact would that have on readiness?

**Respondent:** When such guidelines are in place and put to use, the committee works more efficiently for timely decision-making and resource mobilization towards health service delivery.

**Interviewer:** Among all the practices discussed, which do you think is the best practice for improving readiness?
**Respondent:** I believe coordination is the best practice. When coordination among staff, the management committee, wards, and the executive body is strong, everything can progress smoothly, leading to improved readiness.

**Interviewer:** What steps are necessary to replicate the good practices of this health facility in other facilities?
**Respondent:** We can demonstrate the good practices implemented here to other health facilities. Previously, the health section chief of the nearby Sigash Rural Municipality brought all health facility in-charges here to observe our practices. They analyzed our strengths and discussed with the management team. Similarly, such exchanges can help other facilities adopt best practices. Regular meetings of health facility in-charges to share their strengths would also be beneficial.

**Interviewer:** What should Baitadi District focus on to improve health readiness?
**Respondent:** To improve health facilities, staff unity is crucial. Management committee meetings should be held regularly, as should staff meetings. Coordination between staff, management committees, and municipalities will further enhance readiness.

**Interviewer:** Should the existing policies be implemented effectively, or do you think changes in policies are necessary to improve readiness?
**Respondent:** Regarding policies, the current MSS program is designed for all levels of health facilities, from the smallest to the largest hospitals. If separate standards were developed for lower-level health facilities, it could play a significant role in improving their readiness.

**Interviewer:** Why is medicine availability easier here?
**Respondent:** The executive committee holds meetings to decide on medicine procurement. The municipality ensures timely tenders, so our facility has not faced a shortage of medicines so far.

**Interviewer:** How has internet availability helped in health information management?
**Respondent:** Internet and Wi-Fi have been immensely helpful. They enable timely and accurate recording and reporting. Without the internet, proper data management would not be possible. The internet has supported the eLMIS system, and implementing eHMIS would further improve efficiency.

**Interviewer:** Finally, is there anything I missed asking regarding readiness that you would like to share?
**Respondent:** I believe we covered everything. There’s nothing more to add.

**Interviewer:** Thank you, sir.
**Respondent:** Thank you.

**KII_H**

**Interviewer:** Namaste, sir.
**Respondent:** Namaste.

**Interviewer:** Previously, we conducted a readiness assessment, and this Shrikot Health Post ranked higher in readiness compared to other health facilities. In today’s interview, we would like to discuss the reasons behind this improved readiness. To start, could you tell us your position here and how long you’ve been working?

**Respondent:** My qualification is HA (Health Assistant). Currently, I’m working as AHW and have been here for one year.

**Interviewer:** Why do you think the readiness of this facility is higher compared to others?
**Respondent:** Sir, just having instruments isn’t enough. In some health posts, despite having everything, patients still don’t prefer to visit. It could be due to a lack of proper counseling. If we can’t provide good counseling, that might be one reason. Here, we provide proper counseling to every patient who visits.

**Interviewer:** Let’s focus on the perspective of the health institution rather than the patients. Earlier, I asked questions based on five domains. What do you think contributed to better readiness in those areas?

**Respondent:** Speaking of those domains, I would say that the municipality has been supplying us with the required medicines and equipment as per our requests. First of all, I want to thank the municipality, as it has played a key role. We also have our role, whenever we identify any shortcomings, we immediately make a request, and they supply the needed resources promptly.

**Interviewer:** In some health facilities, NGOs and INGOs also contribute to improving readiness. Has any non-governmental organization supported this Shrikot Health Post?
**Respondent:** Previously, there were some contributions, sir. But recently, no organization has provided direct support. Occasionally, WHO provides advice and support regarding recording and reporting on vaccine-preventable diseases.

**Interviewer:** How is your coordination with the municipality and wards, and how has this contributed to improved readiness?

**Respondent:** One cannot achieve this alone. Good coordination is essential. It’s not enough for health workers to work hard alone. The availability of essential medicines, equipment, and other resources depends on coordination. Our coordination with the municipality and ward is excellent. They have been very supportive in all aspects.

**Interviewer:** What kinds of suggestions do you receive from patients regarding your health services, and how do you gather and address them?

**Respondent:** We have 13 mothers' groups here. We also participate in their meetings and gather suggestions. Additionally, we conduct PHC-ORC sessions, from which we also collect feedback. So far, we haven’t received any major complaints. Two of us stay here at all times, and we provide services 24/7. We never say the facility is closed. This has been appreciated, and we haven’t received any negative comments so far.

**Interviewer:** How often do supervisory visits occur from the municipality or health office, and how do these visits help improve readiness?

**Respondent:** Supervision has been very helpful. Just recently, officials from the health office visited us. A public health inspector from the district and the municipality chairperson also visited. They observed how we deliver services and identified any gaps. When they personally visit and see the shortcomings, they address them and provide supplies. This also motivates the staff.

**Interviewer:** This health institution is connected to the road and located along the main highway. Do you think geographical convenience has contributed to its higher readiness?
**Respondent:** Infrastructure and transportation play a role, sir. The good infrastructure here was easier to establish because of its proximity to the road. It is also convenient for service users, such as for deliveries, as vehicles can directly reach the health facility. Road access also makes it easier for supervisory visits to occur. Additionally, the supply of medicines and other equipment has been more efficient due to road access, enabling timely deliveries.

**Interviewer:** Do you think readiness varies according to the type of health institution?
**Respondent:** In my opinion, it does, sir. Higher institutions have more staff and equipment. On the other hand, some facilities face infrastructure and transportation issues. Looking at it this way, I believe readiness varies.

**Interviewer:** How often are staff trained, and how does this help improve readiness?
**Respondent:** Trainings are conducted periodically. Some staff are new and have more theoretical knowledge than practical experience. Theoretical knowledge alone makes behavior change more difficult. Trainings provide new practical skills, which contribute to improving readiness.

**Interviewer:** We often face budget limitations. If the leadership in the municipality and ward is sensitive to health issues, it helps. How have you found the leadership here, and how has it contributed to readiness?

**Respondent:** In some places, leadership pays little attention to health, focusing instead on other plans. But here, the municipal and ward chairs are actively involved. It’s not just about supplying medicines, buildings are also important, including for PHC–ORC services. Leadership support is crucial for such infrastructure. Here, the leadership has been very supportive whenever needed.

**Interviewer:** How often do staff meetings and management committee meetings occur, and how do routine meetings help improve readiness?

**Respondent:** We hold staff meetings every month to discuss each staff member’s work. We emphasize teamwork, stating that it’s not a one-person job. We discuss achievements, identify gaps, and plan for the next month. Management committee meetings are also held periodically where we present health facility problems and create plans.

**Interviewer:** If staff are motivated, work becomes easier. How motivated are the staff here, and how does it contribute to readiness?

**Respondent:** Motivation brings new energy. It doesn’t just improve readiness; it enhances everything. Even for contract staff, we encourage teamwork, telling them that being on contract doesn’t matter. Everyone supports each other, and the staff here are motivated, which also contributes to readiness.

**Interview:** If management committee guidelines and a citizen charter are in place, what impact would that have on readiness?

**Respondent:** The availability of the HFOMC guideline ensures that there is no ambiguity in decision-making processes. This clarity helps in maintaining accountability and facilitates the smooth flow of operation in the health facility, thereby enhancing readiness to deliver quality health services. It helps the members to understand their role in resource allocation and mobilization when the guideline is available. We have put the citizen charter outside. This has definitely made it easier for service users. Rather than it contributes directly to the readiness of the health facility, I think every health facility that is ready should have one. The municipality provides us with flex print of it. Sometimes children come and tear it down, but we coordinate with the health section and request replacements.

**Interviewer:** We’ve discussed various factors contributing to higher readiness. Among these, what do you think is the most important best practice?

**Respondent:** The most important factor is staff unity. Unity is essential. Sometimes, staff might compare their efforts to others, saying things like, “Why should I do this when someone else didn’t?” or “They took a day off, so I’ll take one too.” I believe we should work as a family. This unity helps improve readiness.

**Interviewer:** What should be done to replicate the good practices of this institution in other facilities?
**Respondent:** I’d say, first, identify and address the specific shortcomings in other facilities. It’s not necessary to do things exactly as we do here; they can implement new ideas. For example, if they lack essential medicines, coordination, or buildings, they should collaborate with the municipality or district to resolve these issues.

**Interviewer:** In the context of Baitadi District, the readiness here is only 66%. What should be done to improve the district’s overall readiness?

**Respondent:** Supervisory visits from higher authorities should be regular, rather than just blaming staff. Everyone should work together like a unified team. I believe this approach will improve readiness.

**Interviewer:** What makes medical supply efficient here?
**Respondent:** For large-scale medical supplies, there’s a tender process, which can sometimes be delayed for various reasons. In such cases, the municipality purchases emergency supplies of medicines to address shortages. They promptly fulfill gaps with emergency procurement.

**Interviewer:** To improve the readiness of health facilities, should current policies be changed, or should we focus on better implementation?
**Respondent:** The policies are fine, sir. They are well-designed.

**Interviewer:** Lastly, regarding readiness, is there anything I missed that you would like to share?
**Respondent:** A key strength here is staff unity, sir. The municipality ensures no shortage of equipment. Leadership also plays a crucial role, when leadership is strong, staff perform well too.

**Interviewer:** Thank you, sir.
**Respondent:** Thank you.

KII_I

**Interviewer:** Sir, Namaste.
**Respondent:** Yes, sir, Namaste.

**Interviewer:** Previously, we assessed the readiness of various health facilities in Baitadi. Among the urban health centers in the district, Gurukhola Urban Health Center has shown good readiness. In this interview, we’ll discuss the reasons behind its high readiness and the facilitators contributing to it. To begin, how long have you been working here? Can you share your work experience and your current position?

**Respondent:** I have been working as a AHW at this urban health center for the past five years.

**Interviewer:** What do you think are the reasons for the good readiness of this health facility?
**Respondent:** The main reasons are, firstly, its location near the road. Additionally, Dasharathchand Municipality has provided the necessary equipment and resources as per the standards. Another reason is our strong teamwork.

**Interviewer:** Some health facilities receive support from various NGOs/INGOs. Has this center received any such support?

**Respondent:** Once, an organization provided us with an inverter. Apart from that, there hasn’t been any other support.

**Interviewer:** How can NGO/INGO support enhance readiness?
**Respondent:** If any organization provides support in areas such as equipment that align with health protocols, readiness improves. It would allow us to deliver better services than we currently do.

**Interviewer:** Coordination is also essential for improving readiness. How is your coordination with the municipality and ward, and how has it contributed to readiness?
**Respondent:** Our coordination is quite good. The required resources have been made available to us, which has positively contributed to readiness.

**Interviewer:** How often do supervision visits occur, and how do they contribute to readiness?
**Respondent:** Officials from Dasharathchand Municipality and the health office visit us for supervision. They provide us with guidance and advice, pointing out our weaknesses and suggesting ways to address them. This has been very helpful for us.

**Interviewer:** Earlier, you mentioned the location near the road as a factor. How has being close to the road contributed to improved readiness?

**Respondent:** Being close to the road has made it easier to bring in all the necessary supplies. Emergency items can be procured within an hour. Referring emergency cases is also more convenient.

**Interviewer:** How do you collect suggestions from service users, and what kind of feedback do you usually receive?

**Respondent:** Service users give us positive feedback and suggestions. They often say, “You’re doing well, and we encourage you to keep improving.”

**Interviewer:** How often do staff receive training, and how has it contributed to improving readiness?
**Respondent:** The municipality and health office provide us with the necessary training. The things we learn in training have been very beneficial when implemented here.

**Interviewer:** If the municipality and ward leadership are sensitive to health issues, it helps. What has been your experience with the leadership here, and how has it contributed to readiness?
**Respondent:** The leadership has been very supportive. The municipality provides us with the required resources whenever needed. The ward also provides as much assistance as they can.

**Interviewer:** How have staff meetings and management committee meetings played a role in improving readiness?

**Respondent:** In staff meetings, we discuss our shortcomings, things that need to be done, and plan for the future. Management committee meetings also occur regularly, where they provide us with advice and guidance. This has been very helpful.

**Interviewer:** What role does staff motivation play in achieving higher readiness here?
**Respondent:** There’s excellent coordination among the staff. We work together and support each other in all situations. Everyone is enthusiastic about their work, which has certainly contributed to improving readiness.

**Interviewer:** We’ve discussed many aspects. Among them, what do you think is the best practice contributing to higher readiness here?
**Respondent:** The timely availability of medicines and other necessary supplies from the municipality, coupled with our efforts to provide quality services, are the main factors, I believe.

**Interviewer:** How can the practices of this health facility be replicated in facilities with lower readiness?
**Respondent:** With proper training arrangements and good supervision providing advice and suggestions, other facilities can also improve.

**Interviewer:** What should be done to improve the overall readiness of health facilities in Baitadi District?
**Respondent:** First, resources and equipment should be made available in all areas as per the guidelines set by the Government of Nepal. This would make it easier for healthcare workers to perform their duties. Additionally, healthcare workers should work efficiently based on their experience and qualifications to improve readiness.

**Interviewer:** Do you think current policies need changes, or should we focus on better implementation?
**Respondent:** The current policies are fine, but further improvements could be made by conducting research with experts to identify and address gaps.

**Interviewer:** Lastly, is there anything else you’d like to share regarding readiness?
**Respondent:** That’s all, sir. We’ve discussed many things. To make it even more effective, we might need to address things we haven’t considered. We’ve been following the guidelines, and with proper supervision and advice, we can achieve even better results.

**Interviewer:** Thank you, sir.
**Respondent:** Thank you.

**KII_J**

**Interviewer:** Sir, Namaste.
**Respondent:** Namaste, sir.

**Interviewer:** During our previous visit, we assessed the readiness of this community health unit. Compared to other community health units, this one has shown better readiness. In this interview, we will discuss the reasons behind this. To start, how long have you been working here, and in what position?

**Respondent:** I have been working as a health assistant for the past four years, sir.

**Interviewer:** Some health facilities receive support from NGOs/INGOs. Has this unit received any support from such organizations?

**Respondent:** So far, no NGOs/INGOs have provided support, sir.

**Interviewer:** If NGOs/INGOs were to provide support, how could it help improve readiness?
**Respondent:** If they supported the physical infrastructure or equipment of this unit, it would help improve readiness.

**Interviewer:** Coordination with the municipality and health section is also important. How are you maintaining coordination, and how has it contributed to readiness?
**Respondent:** Our coordination with the rural municipality and health section is good. The municipality office is also nearby. Whenever we coordinate with the health section for medicines or other necessary items, they respond positively.

**Interviewer:** Supervision is also crucial. How often do supervisory visits take place from higher levels, and how have they contributed to readiness?
**Respondent:** Regarding supervision, the coordinator of the health section lives nearby and visits frequently for supervision. The ward chairperson also visits occasionally. During their visits, they identify weaknesses and provide appropriate advice and suggestions, which have helped improve readiness and made service delivery easier.

**Interviewer:** This health unit is also close to the road and geographically accessible, as well as near the municipality. How has this accessibility contributed to improved readiness?
**Respondent:** Yes, that’s true. Being close to the municipality and the road has made it easier to transport medicines whenever needed. The proximity to the road also facilitates regular supervision visits.

**Interviewer:** How often do staff receive training, and how has it contributed to readiness?
**Respondent:** Trainings are conducted from time to time, sometimes at the health office and sometimes at the training center in Dhangadhi. Implementing the skills learned from these trainings has helped improve readiness here.

**Interviewer:** How do regular management committee and staff meetings help improve readiness?
**Respondent:** We hold staff meetings every month to review the work done in the previous month and plan for the upcoming month. We discuss our weaknesses and areas for improvement. The management committee also holds meetings periodically, where we raise issues from the staff and the health unit’s perspective. We request them to address these issues, and this has helped improve readiness.

**Interviewer:** If the leadership at the municipality or ward level is positive towards health, it makes things easier. What has been your experience with the leadership here, and how has it contributed to readiness?

**Respondent:** We staff hold discussions about how to improve the health unit. For problem-solving, we communicate with the health section and the chairperson. They respond positively and are proactive in addressing the issues we bring to their attention.

**Interviewer:** Finally, is there anything else you’d like to share about readiness?
**Respondent:** Everything has already been covered, sir. Nothing more to add.

**Interviewer:** Thank you, sir.
**Respondent:** Thank you.
